# Connecting Agent-Based Models with High-Dimensional Parameter Spaces to Multidimensional Data Using SMoRe ParS: A Surrogate Modeling Approach

**DOI:** 10.1007/s11538-023-01240-6

**Published:** 2023-12-30

**Authors:** Daniel R. Bergman, Kerri-Ann Norton, Harsh Vardhan Jain, Trachette Jackson

**Affiliations:** 1 Department of Mathematics, University of Michigan, 530 Church Street, Ann Arbor, MI 48109 USA; 2 Computational Biology Laboratory, Computer Science Program, Bard College, 30 Campus Road, Annandale-on-Hudson, NY 12504 USA; 3 Department of Mathematics & Statistics, University of Minnesota Duluth, 1117 University Drive, Duluth, MN 55812 USA

**Keywords:** Agent-based model, Cancer, Model parameterization, Parameter identifiability, Surrogate model

## Abstract

Across a broad range of disciplines, agent-based models (ABMs) are increasingly utilized for replicating, predicting, and understanding complex systems and their emergent behavior. In the biological and biomedical sciences, researchers employ ABMs to elucidate complex cellular and molecular interactions across multiple scales under varying conditions. Data generated at these multiple scales, however, presents a computational challenge for robust analysis with ABMs. Indeed, calibrating ABMs remains an open topic of research due to their own high-dimensional parameter spaces. In response to these challenges, we extend and validate our novel methodology, Surrogate Modeling for Reconstructing Parameter Surfaces (SMoRe ParS), arriving at a computationally efficient framework for connecting high dimensional ABM parameter spaces with multidimensional data. Specifically, we modify SMoRe ParS to initially confine high dimensional ABM parameter spaces using unidimensional data, namely, single time-course information of in vitro cancer cell growth assays. Subsequently, we broaden the scope of our approach to encompass more complex ABMs and constrain parameter spaces using multidimensional data. We explore this extension with in vitro cancer cell inhibition assays involving the chemotherapeutic agent oxaliplatin. For each scenario, we validate and evaluate the effectiveness of our approach by comparing how well ABM simulations match the experimental data when using SMoRe ParS-inferred parameters versus parameters inferred by a commonly used direct method. In so doing, we show that our approach of using an explicitly formulated surrogate model as an interlocutor between the ABM and the experimental data effectively calibrates the ABM parameter space to multidimensional data. Our method thus provides a robust and scalable strategy for leveraging multidimensional data to inform multiscale ABMs and explore the uncertainty in their parameters.

**Supplementary Information:**

The online version contains supplementary material available at 10.1007/s11538-023-01240-6.

## Introduction

Biological experiments typically involve numerous independent, yet potentially causally correlated variables, measurement modalities, and experimental conditions (Krzywinski and Savig 2013). These multidimensional data sets offer tremendous potential for advancing our knowledge of the world around us, but they also present inherent challenges. For example, complex data types are only partially tractable with current mathematical, statistical, and computational techniques (Argelaguet et al. 2021; Hariri et al. 2019; Rockne et al. 2019; Qiu et al. 2021). Furthermore, available experimental data are often limited, noisy, coarse-grained, and lack spatial resolution, exacerbating these challenges.

Of the variety of mathematical and computational modeling approaches that exist to describe, analyze and interpret complex data, agent-based models (ABMs) have emerged as a powerful tool for understanding the interconnected molecular, cellular, and microenvironmental dynamics in health and disease (Norton et al. 2019; Shuaib et al. 2016; Badham et al. 2018; Rikard et al. 2019; West et al. 2023). ABMs characterize populations as individual agents with distinct properties and behaviors, enabling interactions with their local environment, which includes other agents, to produce emergent global phenomena. This unique approach allows ABMs to capture both the interconnectedness and heterogeneity in biological, environmental, and social systems across multiple time and spatial scales.

To make meaningful, reliable quantitative predictions and to gain mechanistic insights, ABMs must be integrated with real-world data through model parameterization and calibration (Eisenberg and Jain 2017; Byrne 2010; Gatenby and Maini 2003). However, the inherent stochastic nature and extensive computational demands involved in simulating large agent populations make it challenging to explore ABM parameter spaces thoroughly. Monte Carlo simulations, genetic algorithms, and Bayesian methods have all been used to estimate ABM parameters (Broniec 2021; Calvez and Hutzler 2005; Klank et al. 2018; Lee et al. 2015; Nardini et al. 2021). However, these methods suffer from high computational expenses, reliance on prior knowledge, and limited applicability beyond specific parameter regimes (Nardini et al. 2021; Klank et al. 2018).

New, thoughtfully developed mathematical methods are desperately needed to calibrate and validate computationally complex models with real-world multidimensional data, which can span different time and/or spatial scales. In this paper, we validate and extend our new approach, Surrogate Modeling for Reconstructing Parameter Surfaces (SMoRe ParS) (Jain et al. 2022), which is a first-of-its-kind method that leverages explicitly formulated surrogate models to bridge the computational divide between ABMs and experimental data. Surrogate models, also known as meta-models or response surface models, are a valuable tool for reducing the computational burden associated with complex models—like ABMs—enabling more efficient analysis (Alizadeh et al. 2020; Asher et al. 2015; Blanning 1975; Pietzsch et al. 2020). They are often statistical or machine learning models that serve as a substitute for the original model to reduce the computational cost of making predictions or conducting optimization tasks. Engineering and weather forecasting applications commonly employ surrogate models (see Palar et al. 2019; Schultz et al. 2021 for reviews), and the use of machine learning algorithms to generate surrogate models that do not have a closed form is now a favored approach. However, the underlying mechanistic detail of the phenomena being modeled is not retained with these “black box” surrogate models.

Our approach uses an explicitly formulated, data-informed, and easy-to-simulate surrogate model to quantify the relationship between computationally complex model inputs and surrogate model parameters, and between surrogate model parameters and real-world data. Acting as a bridge connecting difficult-to-estimate ABM inputs with noisy real-world data, surrogate model parameters facilitate the calibration and uncertainty quantification of ABM parameters, directly aligning them with given experimental data. We have previously demonstrated the potential of our approach in a limited proof-of-concept (Jain et al. 2022), where we used limited time-course experimental data to calibrate a small subset of parameters (that is, a low dimensional parameter space) in an ABM of vascular tumor growth. We now adapt SMoRe ParS so that it can first constrain high dimensional ABM parameter space using unidimensional (single time-course) data, taking in vitro cancer cell growth assays as our application of choice. We then extend our method to constrain parameters in a more complex ABM with multidimensional (multiple time-courses at different biological scales) data, taking in vitro cancer cell inhibition assays with the chemotherapeutic compound oxaliplatin as our application of choice. We validate our method for each case by comparing the match to experimental data of ABM simulations with SMoRe ParS and directly inferred parameters using several statistical metrics. By doing so, we demonstrate that explicitly formulated surrogate models, informed by both experimental data and ABM output, enable high dimensional ABM parameter spaces to be constrained using multidimensional data. Our approach provides a novel, scalable method for linking ABMs with high-dimensional parameter spaces to multidimensional data.

## Methods

In this section, we describe the various computational modeling approaches, mathematical analysis techniques, and experimental data used in this paper. We begin with a description of the experimental data used to calibrate our models. We then describe our agent-based model (ABM) formulation followed by an overview of the SMoRe ParS method. Next, we develop a surrogate model (SM) using ordinary differential equations, and discuss ABM output generation for calibrating SM parameters. We then discuss SM parameterization, and parameter uncertainty quantification and identifiability. Finally, we describe how ABM parameters are inferred using SMoRe ParS as well as directly. These steps are described in detail in the following subsections.

### Experimental Data

**Fig. 1:**
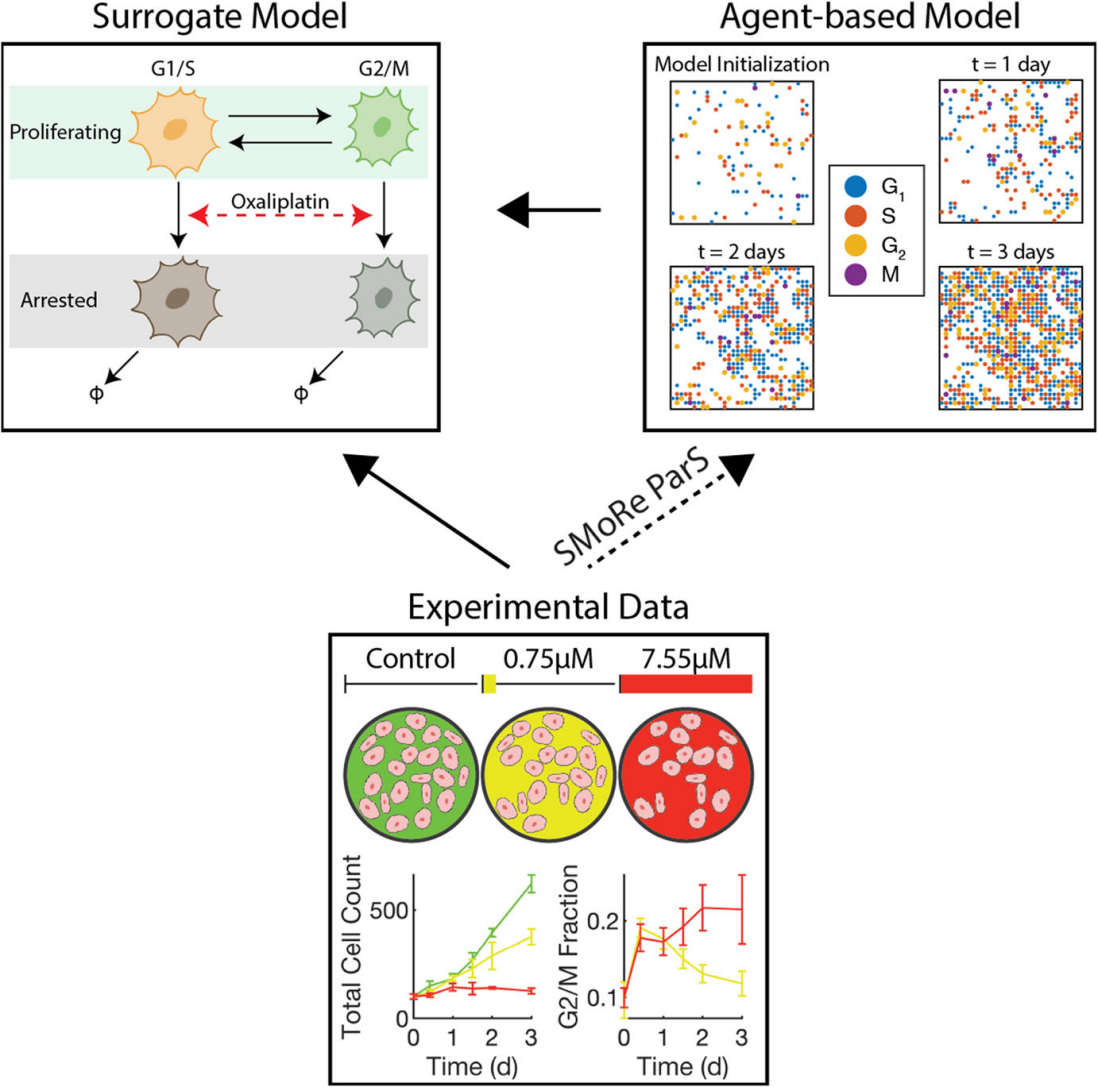
Schematic of using SMoRe ParS to infer ABM input parameters from experimental data via a surrogate model. The solid arrows connecting Experimental Data and the Agent-based Model to the Surrogate Model represent the direction of information flow in the first few steps of SMoRe ParS. Green (control), yellow (0.75$\upmu$M oxaliplatin) and red (7.55$\upmu$M oxaliplatin) colors refer to the experimental dosing regimes that generated the experimental data. The green total cell count curve in the experimental data box corresponds to the control case, for which the experiments did not collect cell cycle distribution information, hence the missing curve in the G2/M fraction plot

The impact of the chemotherapeutic agent oxaliplatin on the growth of the SNU-1 human gastric cancer cell line has been previously evaluated through a series of experiments detailed in Jang et al. (2002). Briefly, cells were cultured in a control medium for 24 h, followed by exposure to either two different constant concentrations of oxaliplatin (0.75$\upmu$M and 7.55$\upmu$M) or no drug (control) for 72 h. Viable cell counts were recorded at five specific time points, resulting in three time-series data sets, for cell growth and inhibition. Additionally, flow cytometry yielded cell cycle distributions at each time point for the two treatment time courses. These data are shown in Fig. 1. We note that total cell numbers were normalized to an initial condition of 100 cells to enable direct comparison with simulated (ABM) data.

### Agent-Based Model (ABM) Formulation

Our agent-based model is an extension of a 2D, on-lattice birth-death-migration model (Bergman and Jackson 2023). At initialization, agents are seeded uniformly throughout a square microenvironment. The size of the microenvironment is determined by the carrying capacity $K_A$, a parameter we vary. For a given carrying capacity, we set the length of the microenvironment as $l=\lceil \sqrt{K_A}\rceil$ and the width as $w=\lceil K_A/l \rceil$, where $\lceil \cdot \rceil$ represents the ceiling function. At discrete time steps, agents update by advancing through the cell cycle, undergoing apoptosis, or moving. In simulations with chemotherapy, the drug is applied uniformly in time and space throughout the simulation. This mimics the experimental conditions in the cell growth inhibition assays described in the previous subsection. In what follows, an “open” lattice site within the microenvironment refers to an unoccupied site. Neighboring lattice sites refer to the von Neumann neighborhood, that is, the four nearest neighbor sites. The ABM algorithm is summarized in a flowchart, shown in Figure S1 in the Supplementary Information. Key steps of the algorithm are detailed below.

#### Progression Through Cell Cycle

Each agent or cell advances through four stages of the cell cycle in order: G1, S, G2, and M with transition rates, $\{\rho _{\text {G1}\rightarrow \text {S}},\rho _{\text {S}\rightarrow \text {G2}},\rho _{\text {G2}\rightarrow \text {M}},\rho _{\text {M}\rightarrow \text {G1}}\}$. When a cell advances from M back to G1, it can proliferate into an open neighboring lattice site, depending on the extent of contact inhibition on the cell. Specifically, if the number of open lattice sites is below a threshold, $T_\text {con}$, the cell returns to G1 without undergoing mitosis. Otherwise, mitosis occurs and two daughter cells are introduced in the G1 phase.

#### Cell-Cycle Arrest

Oxaliplatin is a cell cycle non-specific, platinum-based compound used to treat a variety of cancer types (Baker 2003). Oxaliplatin interferes with the DNA replication process by covalently binding to DNA molecules. The formation of DNA cross-links and adducts causes DNA damage that is recognized at the G1/S or G2/M checkpoints (Kastan and Bartek 2004), ultimately leading to cell-cycle arrest.

Following (Eisenberg and Hayashi 2014), the probabilities $$p_{\text {arrest},i}$$ ($$i = $$G1, G2) of arrest during these two transitions are assumed to be increasing and saturating functions of the administered drug concentration, with half-saturation constant $$\gamma _1$$ and Hill exponent *a*. The maximum possible probability of arrest $$r_i$$ depends on the affected cell’s current cell-cycle phase ($$i = $$G1, G2). That is,

1 $$\begin{aligned} p_{\text {arrest},i}= & {} r_i\frac{C^a}{\gamma _1^a+C^a},\quad i=\text {G1},\text {G2} \end{aligned}$$

#### Apoptosis

The severity and repairability of oxaliplatin-induced DNA damage determines whether or not arrested cells undergo apoptosis (Alcindor and Beauger 2011; Raymond et al. 2002). However, for simplicity we assume that once arrested, cells will ultimately undergo cell death with no possibility of recovery. Further, arrested cells neither advance through the cell cycle nor move. When a cell undergoes apoptosis, it is removed from the simulation after all other updates have been carried out.

The rate of cell apoptosis $p_\text {apoptosis}$ is assumed to be an increasing and saturating function of drug concentration, with half-saturation constant $\gamma _2$, Hill exponent *b* and maximum rate of cell death $\delta _p$ (Eq. 2). That is,

2 $$\begin{aligned} p_\text {apoptosis}= & {} \delta _p \frac{C^b}{\gamma _2^b+C^b} \end{aligned}$$

#### Migration

Cells move to neighboring lattice sites at a constant rate, *s*. When a cell moves, it selects randomly from open neighboring lattice sites, remaining stationary if there are none.

### Surrogate Model for Reconstructing Parameter Surfaces (SMoRe ParS)

**Fig. 2:**
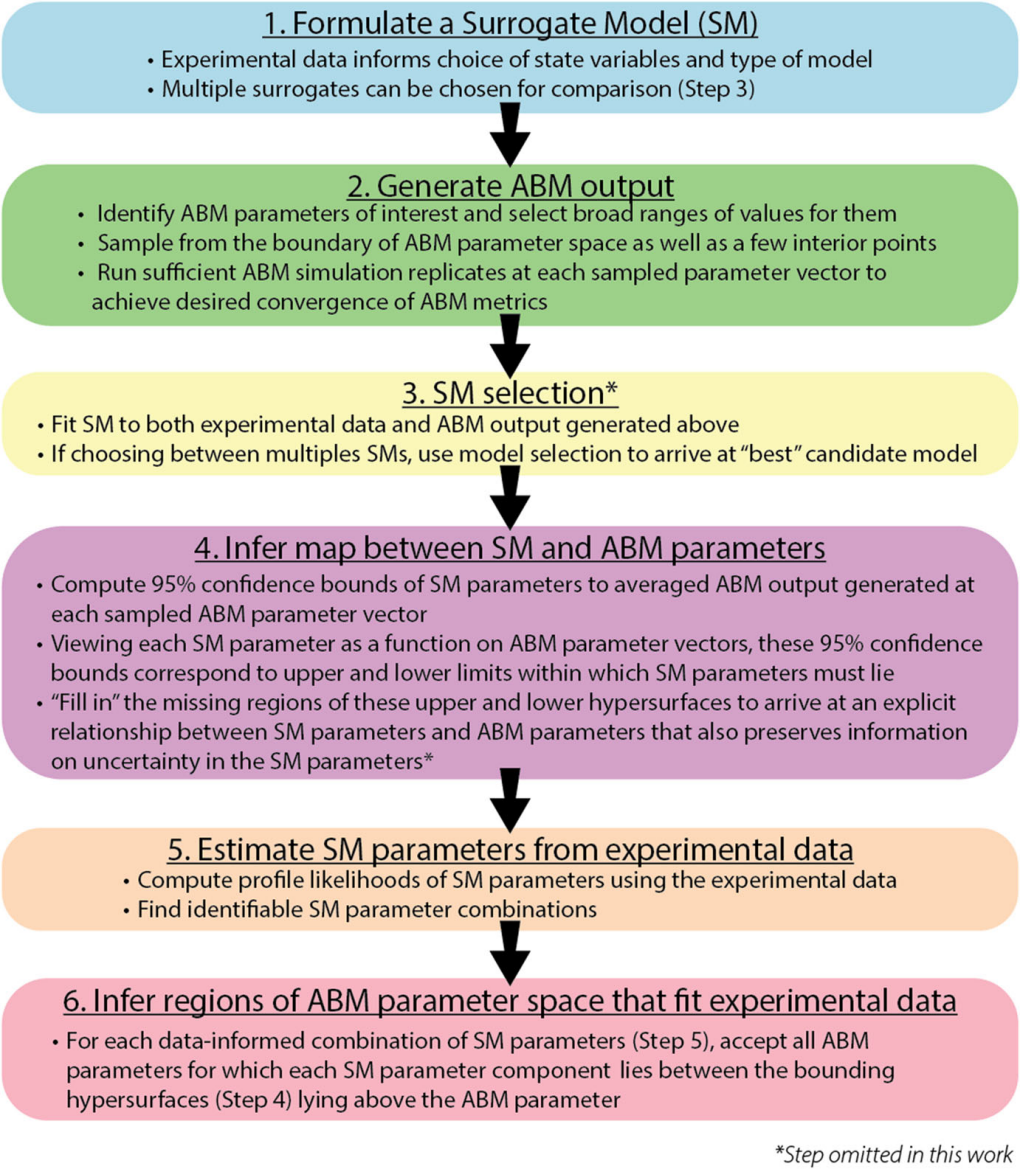
Flowchart of the SMoRe ParS algorithm

We have proposed a first-of-its-kind method (Jain et al. 2022) that leverages explicitly formulated surrogate models for linking computationally complex models such as ABMs and noisy, sparse experimental data (see Fig. 2). Surrogate Modeling for Reconstructing Parameter Surfaces (SMoRe ParS) is implemented as described below for a given ABM and experimental data:

1. Use data to inform surrogate model variables and formulation (e.g., ODE, PDE, Boolean etc.), and arrive at one or more candidate model(s) (see Sect. 2.4).
2. Select a subset of ABM parameters to constrain from the experimental data and generate ABM output for a broad range of values of these parameters (see Sect. 2.5).
3. If necessary, perform model selection to choose between several potential surrogate model candidates by testing their ability to fit both the experimental data and the ABM output.
4. Reconstruct surrogate model parameter surfaces from ABM output by inferring a quantitative relationship between each of the surrogate model input parameters and the selected ABM parameters. This is done by quantifying uncertainty when fitting surrogate model parameters to ABM output that was generated in Step 2 (see Sect. 2.6).
5. Arrive at identifiable combinations of surrogate model input parameters from real-world data by performing a practical identifiability analysis (see Sect. 2.6).
6. Infer regions of ABM parameter space that correspond to real-world data by overlaying the ranges of data-derived SM parameters in the previous step onto the inferred relationship between surrogate model parameters and ABM parameters found in Step 4. This yields regions of ABM parameter space that correspond to experimental data (see Sect. 2.7).

For more details on how to implement SMoRe ParS, we refer the reader to Jain et al. (2022).

### Surrogate Model Formulation

The time-course experimental data taken from Jang et al. (2002) suggests an ordinary differential equation (ODE) formulation for the surrogate model, with cells compartmentalized depending on their stage within the cell cycle.

**Control (untreated) surrogate model equations**: Governing equations for the numbers of cells in G1/S phase ($N_{1S}$) and G2/M phase ($N_{2M}$) are derived as follows. Cells in G1/S phase of the cell cycle enter G2/M on completion of DNA synthesis at a rate $\lambda$. From G2/M, cells are assumed to undergo cell division at a rate $\alpha$ resulting in two new daughter cells in the G1/S phase. As cell numbers increase, free space decreases and cells are more likely to enter quiescence (Norton and Popel 2014). Our model takes into account this space-limited growth of cells in the following manner. As in the ABM formulation, cells in G2/M are less likely to divide into two daughter cells if they experience contact inhibition. Consequently, in our model the rate of producing two daughter cells decreases from a maximum value of $2 \alpha$ to simply $\alpha$ as the total number of cells increases, with no division possible when the total number of cells reach the carrying capacity *K* of the culture dish. That is, cells unable to divide because of crowding simply re-enter the G1/S phase. Combining these processes, we arrive at the following equations governing cell growth in the absence of treatment.

3 $$\begin{aligned} \frac{dN_{1S}}{dt}= & {} -\lambda N_{1S} + \alpha \left( 2 - \frac{N_{1S}+N_{2M}}{K}\right) N_{2M}, \end{aligned}$$

4 $$\begin{aligned} \frac{dN_{2M}}{dt}= & {} \lambda N_{1S} - \alpha N_{2M}. \end{aligned}$$

**Treatment surrogate model equations**:

To simulate the impact of oxaliplatin on cancer cells, we introduce two additional compartments, $A_{1S}$ and $A_{2M}$, to the control model, representing cells arrested in G1/S and G2/M, respectively. Upon administration of oxaliplatin, cells in the G2/M and G1/S phases undergo cell-cycle arrest at drug dose-dependent rates. These rates are taken as increasing and saturating Hill-functions of the administered drug concentration *C*, with $\beta _{2M}$ representing the maximum arrest rate from G2/M and $\beta _{1S}$ from G1/S. Following Eisenberg and Jain (2017), we assume that the half-saturation constant $\kappa _{\beta }$ and Hill-coefficient *m* are the same in both cases.

From the arrested state, cells can undergo apoptosis with studies showing a linear correlation between cell cytotoxicity and the amount of platinum bound to DNA (Siddik 2003). Hence, the rate of cell death from either arrested compartment is assumed to be an increasing and saturating function of drug concentration, with $\delta$ representing the maximum death rate and $\kappa _{\delta }$ the half-saturation constant. As in the ABM formulation, we do not allow arrested cells to recover to the proliferating pool.

Combining these processes, we arrive at the following equations that govern the response of cancer cells to treatment with oxaliplatin.

5 $$\begin{aligned} \frac{dN_{1S}}{dt}= & {} -\lambda N_{1S} + \alpha \left( 2 - \frac{N}{K}\right) N_{2M} - \beta _{{1S}} \frac{C^m}{\kappa _{\beta }^m + C^m} N_{1S}, \end{aligned}$$

6 $$\begin{aligned} \frac{dN_{2M}}{dt}= & {} \lambda N_{1S} - \alpha N_{2M} - \beta _{{2M}} \frac{C^m}{\kappa _{\beta }^m + C^m} N_{{2M}}, \end{aligned}$$

7 $$\begin{aligned} \frac{dA_{1S}}{dt}= & {} \beta _{{1S}} \frac{C^m}{\kappa _{\beta }^m + C^m} N_{1S} - \delta \frac{C}{\kappa _{\delta } + C} A_{1S}, \end{aligned}$$

8 $$\begin{aligned} \frac{dA_{2M}}{dt}= & {} \beta _{{2M}} \frac{C^m}{\kappa _{\beta }^m + C^m} N_{2M} - \delta \frac{C}{\kappa _{\delta } + C} A_{2M}, \end{aligned}$$

where $N = N_{1S}+N_{2M}+A_{1S}+A_{2M}$ is the total number of alive cells.

A list of SM parameters included in our analysis for the control and treatment models is given in Table  1.

**Table 1:** List of SM parameters

Control parameters		Treatment parameters	
Parameter	Meaning	Parameter	Meaning
*K*	Carrying capacity	$\beta _{1S}$	Maximum arrest rate from G1/S
$\lambda$	G1/S $\rightarrow$ G2/M transition rate	$\beta _{2M}$	Maximum arrest rate from G2/M
$\alpha$	G2/M $\rightarrow$ G1/S transition rate	$\kappa _{\beta }$	EC50 of arrest rates
		$\delta$	Maximum rate of cell apoptosis
		$\kappa _{\delta }$	EC50 of apoptosis rate

### ABM Parameter Space and Output Generation

A list of ABM parameters chosen for implementing SMoRe ParS is given in Table  2.

**Table 2:** List of ABM parameters

Control parameters		Treatment parameters	
Parameter	Meaning	Parameter	Meaning
$K_A$	Carrying capacity	$r_{G1}$	Arrest in G1$\rightarrow$S transition
$T_\text {con}$	Contact inhibition	$r_{G2}$	Arrest in G2$\rightarrow$M transition
*s*	Migration rate	$\gamma _1$	EC50 of both arrest rates
$\rho _{\text {G1}\rightarrow \text {S}}$	G1 $\rightarrow$ S transition	$\delta _p$	Apoptosis rate of arrested cells
$\rho _{\text {S}\rightarrow \text {G2}}$	S $\rightarrow$ G2 transition	$\gamma _2$	EC50 of apoptosis rate
$\rho _{\text {G2}\rightarrow \text {M}}$	G2 $\rightarrow$ M transition		
$\rho _{\text {M}\rightarrow \text {G1}}$	M $\rightarrow$ G1 transition		

ABM output was generated on a regular grid in parameter space, as follows. For the control model, we vary seven parameters choosing three values each (referred to as ‘low’, ‘medium’ and ‘high’), for a total of $3^7$ parameter vectors. For each parameter combination, the ABM was simulated six times to get meaningful average behavior.

We vary five ABM parameters in the treatment model, once again choosing three values each, for a total of $3^5$ parameter vectors. Because we have data for only two non-zero drug concentrations and the Hill function response of cells to oxaliplatin in Eqs. 1 and 2 has three parameters, we hold the Hill coefficients fixed at $a=1$ and $b=1$. For the treatment model, all control parameters were set to the average value of those accepted from the control study. The ABM was simulated for each of the three doses represented in this study: control, 0.75 ${\upmu \hbox {M}}$, and 7.55 ${\upmu \hbox {M}}$. For each of these $3\times 3^5$ conditions, we ran six simulations to get meaningful average behavior.

### Surrogate Model Parameter Calibration, Uncertainty Quantification and Identifiability

Surrogate model parameter values were selected to achieve best fits of tumor cell counts (total number and numbers of cells in G1/S and G2/M) to experimental data as well as ABM output, employing a weighted least squares approach. This was implemented in Matlab using the fmincon constrained optimization tool in conjunction with the built-in ode45 ordinary differential equations solver.

The profile likelihood method described in Venzon and Moolgavkar (1988) was implemented for inferring uncertainty information for estimated parameters. This approach involves “profiling” each estimated parameter by fixing it across a range of values while estimating the remaining parameters for each fixed value. The maximum likelihood function value for each such parameter value generates a likelihood profile. Confidence bounds were calculated using likelihood profiles and a specified threshold, taken here at a 95% confidence level. Additionally, SM parameter profiles were used to arrive at practically identifiable combinations of estimated parameters when fitting to experimental data following the approach described in Eisenberg and Jain (2017).

### ABM Parameterization Using SMoRe ParS

Let $S\subset {\mathbb {R}}^n$ be a set of experimental data-informed SM parameter values, where *n* is the number of SM parameters being fit. For instance, *S* could simply be the singleton set comprising the best fit values of the SM parameters to data. However, this choice would not propagate the uncertainty in SM parameters from the experimental data to the inferred ABM parameter region. Or *S* could be the set arrived at in the previous section (Methods Sect. 2.6), which accounts for the uncertainty in SM parameter values stemming from the noisy and sparse nature of the data. The final step of SMoRe ParS is implemented as follows to arrive at desired ABM parameters that fit the experimental data, using the set *S* as an interlocutor.

**Fig. 3:**
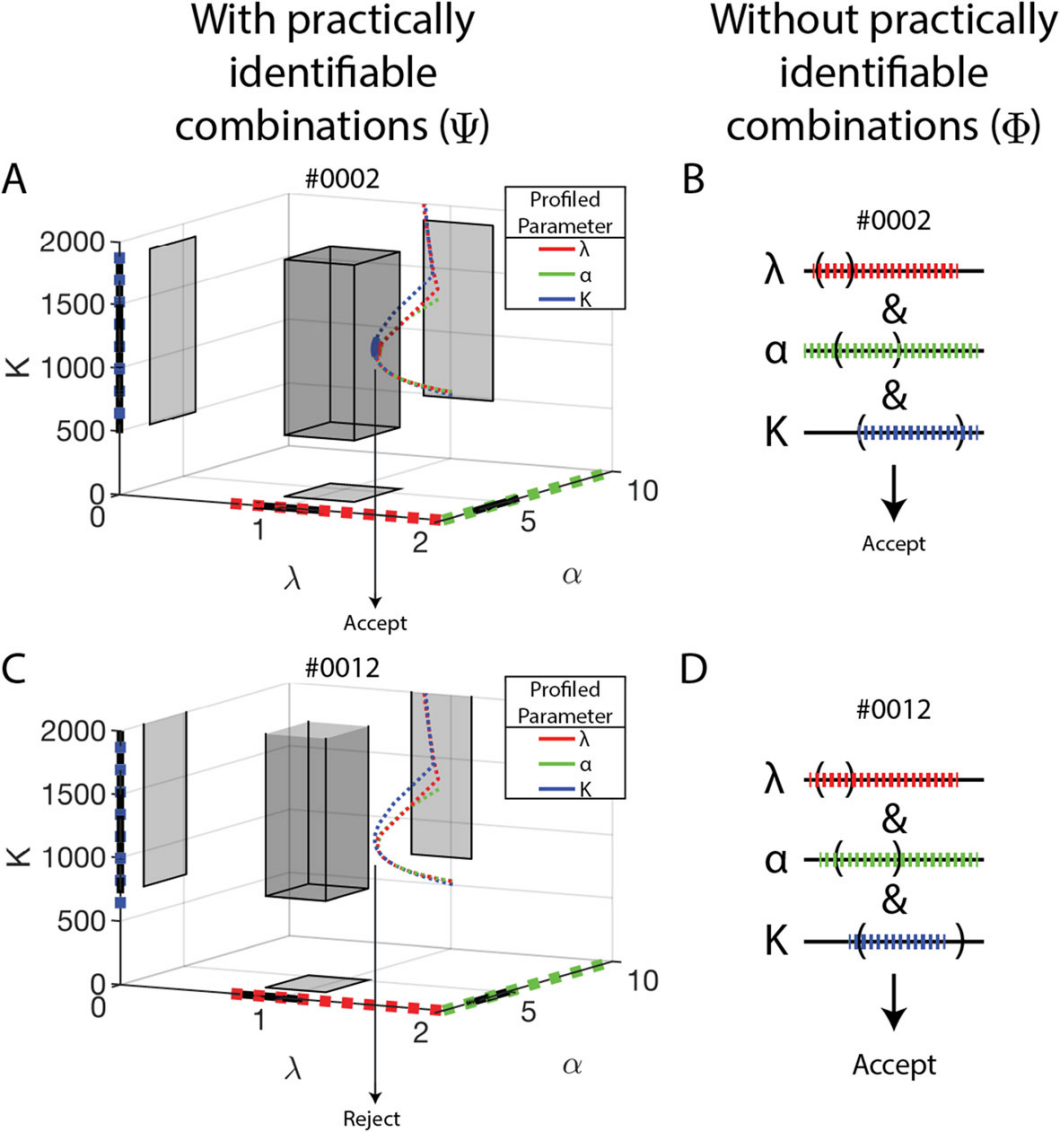
Geometric interpretation for how SMoRe ParS accepts or rejects parameters with (left column, $\Psi$) or without (right column, $\Phi$) using practically identifiable parameter combinations. Each row corresponds to the same single ABM parameter vector: #0002 (top row, accepted by both methods) and #0012 (bottom row, accepted by $\Phi$ only). ABM output at each is used to profile SM parameters, and the 95% confidence intervals (thick lines on each axis) are used to define a hyperrectangle in SM parameter space (shaded box in **a**, **c**). The light grey top of the box in C signifies that *K* is not constrained from above for that ABM parameter vector. The SM parameters are also profiled using the experimental data, and in this case parameter combinations are observed. The paths of each of these three profiles through 3D parameter space are shown in red ($\lambda$), green ($\alpha$), and blue (*K*) dotted curves in **a**, **c**. The projections of these profiles onto their corresponding axes are shown as dashed lines of the corresponding color. Using practical identifiable combinations (see Sect. 3.5), SMoRe ParS accepts #0002 (**a**)–because at least one of these three profiles passes through the shaded box–and rejects #0012 (**c**)–because none of the three pass through the shaded box. Using only the confidence intervals (**b**, **d**), that is, using $\Phi$, SMoRe ParS additionally accepts #0012 because all three pairs of intervals overlap as shown on the right

For each ABM parameter vector at which ABM output was generated, the 95% confidence bounds of the SM parameters yield hyperrectangles or orthotopes in SM parameter space. That is, for a specified SM parameter, each corner of a hyperrectangle is a point on one of the upper or lower bounding hypersurfaces for that SM parameter, at that ABM parameter vector. Figure 3 shows two such hyperrectangles in 3-D control SM parameter space (*n* = 3). A given ABM parameter vector is accepted by SMoRe ParS if and only if this box has non-empty intersection with the set *S*.

In the analysis presented here, we construct this set of experimental data-informed SM parameters in two distinct ways. Let $\Phi \subset {\mathbb {R}}^n$ be the Cartesian product of the experimental data-informed 95% confidence intervals for each SM parameter. Figure 3a, b shows these intervals for the 3-D control SM case as dashed, colored lines on each coordinate axis. Alternatively, let $\Psi \subset {\mathbb {R}}^n$ be the union of the experimental data-informed practically identifiable combinations of SM parameters, shown for the 3-D control SM case as (approximately) coincident colored curves in Fig. 3a, b. $\Phi$ and $\Psi$ are related in that the projection of each colored path defining $\Psi$ onto its color-matched axis is the corresponding factor in the Cartesian product defining $\Phi$. Both are valid choices for *S*.

For each ABM parameter vector, we check whether its corresponding 95% confidence hyperrectangle in SM parameter space has non-empty intersection with the above-defined sets. For instance, for the 3-D control SM case, ABM parameter #0002 is accepted using either $\Psi$ (Fig. 3a) or $\Phi$ (Fig. 3b), whereas ABM parameter #0012 is accepted using $\Phi$ (Fig. 3d) but rejected using $\Psi$ (Fig. 3c). Empirically, we see that ABM parameters accepted using $\Phi$ is a superset of those accepted using $\Psi$. A parameter that is rejected in both frameworks is shown in the Supplementary Figure S2.

Unless otherwise stated, SMoRe ParS is implemented using $\Psi$, that is data-informed practically identifiable combinations of SM parameters.

### ABM Parameter Calibration by Direct Method

Rejection sampling, also called the accept-reject method (Olken and Rotem 1995), was employed to directly calibrate ABM parameters from the experimental data. This was implemented as follows. At each ABM parameter vector on the sampled grid, the mean time series were compared to the experimental data using the same objective function as when comparing the SM to the data. The resulting RSS values were ordered and the lowest RSS values were then accepted with the threshold being set based on the number accepted by SMoRe ParS.

## Results

In this section, we describe the results of our computational study. We begin with demonstrating how well SMoRe ParS constrains high-dimensional ABM parameter spaces using uni-dimensional data in the form of cell number time-courses taken from in vitro cancer cell growth assays. We next constrain a high-dimensional parameter space in a more complex ABM using multi-dimensional (multiple time-courses at different biological scales) data that describes tumor response to chemotherapy. In each case, we demonstrate the suitability of our surrogate model choice and show that SMoRe ParS successfully infers ABM parameter spaces that reproduce the experimental data.

### Unidimensional Experimental Data and the ABM Output Successfully Constrain Surrogate Model Parameter Space

**Fig. 4:**
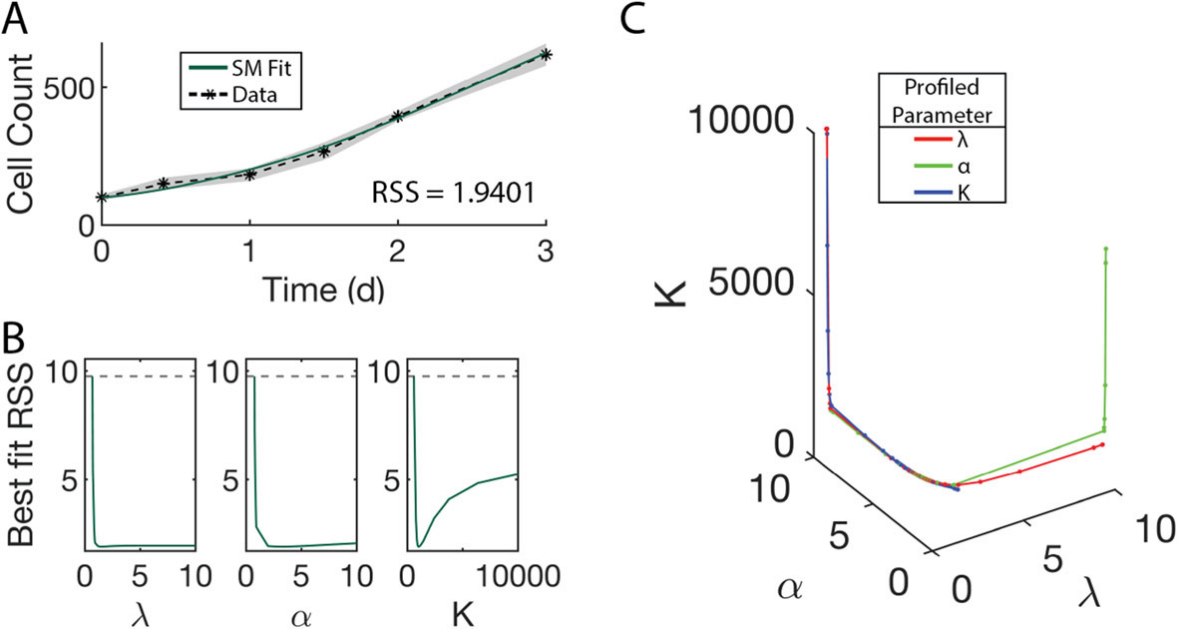
Constraining SM parameters from control arm of experimental data. **a** SM best fit to experimental data. **b** Likelihood profiles of all SM parameters from experimental data. **c** Exploring parameter combinations of the SM fit to the experimental data. The color of the curve corresponds to the parameter being profiled

A key early step in the successful implementation of SMoRe ParS is arriving at a surrogate model (SM) that is well-suited for the experimental data. Our SM for the control (no treatment) case (Eqs. 3 and 4) has three free parameters, $\lambda$, $\alpha$ and *K*. Figure 4a shows a comparison between the time courses of experimental control cell count versus SM-generated output produced using best fit values of $\lambda$, $\alpha$, and *K*. The SM-generated output closely match the experimental data, lying within one standard deviation, with a weighted residual sum of squares (RSS) of 1.9401. We further use profile likelihoods to calculate 95% confidence bounds for each of the SM parameters from noise in the experimental data (Fig. 4b), and conclude that the SM parameters are identifiable from below. Viewing these profiles as paths in SM parameter space, we observe that the three SM parameters are in a single practically identifiable combination, as evidenced by overlapping curves shown in Fig. 4c.

**Fig. 5:**
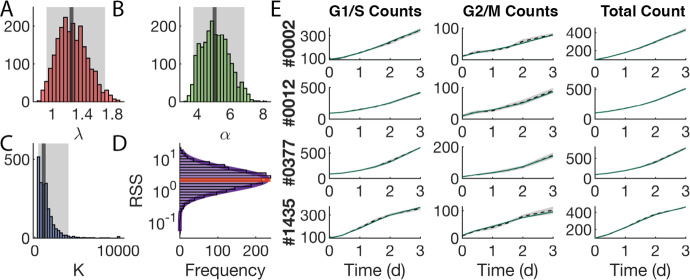
Fitting the SM to each ABM parameter vector output. **a**–**c** Distribution of best fit values of SM parameter $\lambda$ (**a**), $\alpha$ (**b**), and *K* (**c**) to the ABM output. Vertical lines indicates median values. Shaded area indicates the interquantile range. **d** Distribution of RSS values from the best fit of the SM to ABM output at each ABM parameter vector. Orange line indicates the RSS value for the best fit of the SM to the experimental data. **e** Sampling of four ABM parameter vectors (rows) and the fit of the SM to ABM-generated cell counts in the two compartments and ABM-generated total cell count, for a specific choice of ABM parameters (bold numbers on the left)

Next, we fit the control SM parameters to averaged ABM output generated at each of the $3^7$ sampled parameter vectors and plot the distributions for each of the three parameters (Fig. 5a–c). In contrast to when SM parameters were constrained from experimental data, here we also use ABM-generated cell compartment time courses in addition to total cell count time courses when fitting SM parameters. The inferred distributions for $\lambda$ and $\alpha$ are roughly Gaussian (Fig. 5a, b), suggesting that these parameters have a well-constrained range of values that effectively encapsulates the entire ABM output variability. The distribution for *K* is skewed right with several outliers at the user-imposed upper bound of 10,000 cells (Fig. 5c). For these outliers, the value of *K* has little impact on the quality of fits (data not shown).

In order to evaluate the accuracy of the fits, we plot the RSS values of each of the SM fits to the ABM output and of SM fit to the data (Fig. 5d). The RSS values of SM parameters fit to ABM output follow a log-normal distribution (Fig. 5d, purple curve), and are comparable to the RSS from fitting the SM to experimental data (Fig. 5d, orange line). Furthermore, we plot illustrative time series for G1/S and G2/M cell counts of the SM model (in green) and the ABM output (dotted line) with standard deviation shown in gray (Fig. 5e). The time series trajectories for the SM demonstrate good fits to the ABM output in both the G1/S and G2/M compartments.

### SMoRe ParS Creates n-Dimensional Hypersurfaces Bounding each SM Parameter

**Fig. 6:**
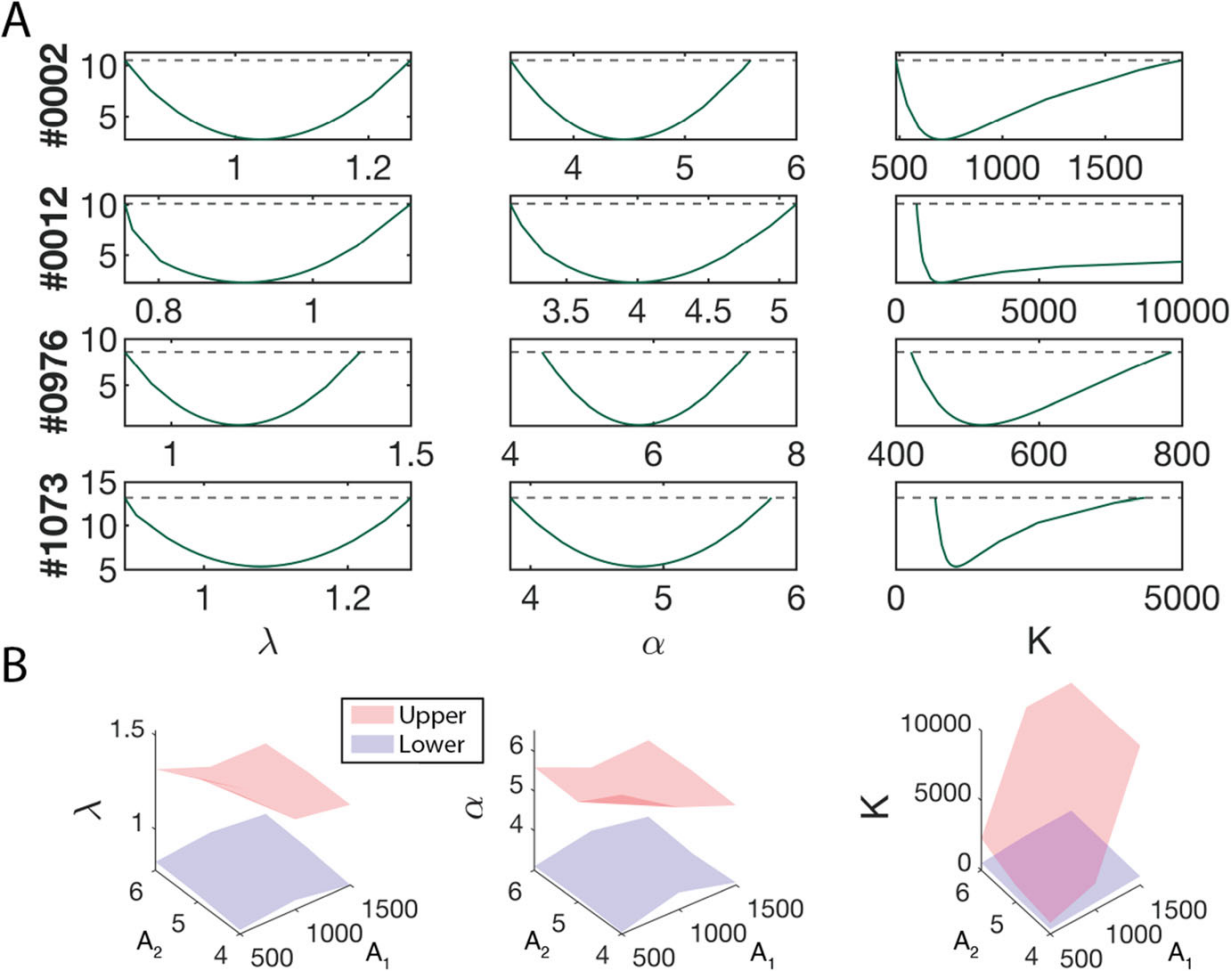
Constraining SM parameters for each ABM parameter vector. **a** Profile likelihoods for SM parameters $\lambda$ (left column), $\alpha$ (middle column), and *K* (right column) using ABM output from four ABM parameter vectors (varied by row). **b** Cross sections of the 7-dimensional lower (blue) and upper (red) bounding hypersurfaces cut parallel to the first two ABM parameter dimensions (Color figure online)

We use the profile-likelihood method to arrive at 95% confidence bounds for each of the SM parameters that were fit to (averaged) ABM output generated at each ABM parameter vector (Fig. 6a). Now, $\lambda$ and $\alpha$ are typically identifiable, unlike when we fit these parameters to experimental data (Fig. 4b). This is because we also use G1/S and G2/M counts when fitting to ABM output, these values not being available in the control experimental data. For a given SM parameter, its 95% confidence bounds correspond to discrete points on the upper and lower 95% confidence hypersurfaces that lie over 7-dimensional ABM parameter space. As an illustration, Fig. 6b shows a cross section of these surfaces cut parallel to the first two ABM parameter dimensions. For this region of ABM parameter space, we are 95% confident that the SM parameters lie in between these hypersurfaces.

### SMoRe ParS Infers ABM Parameter Spaces that Successfully Reproduce the Experimental Data

**Fig. 7:**
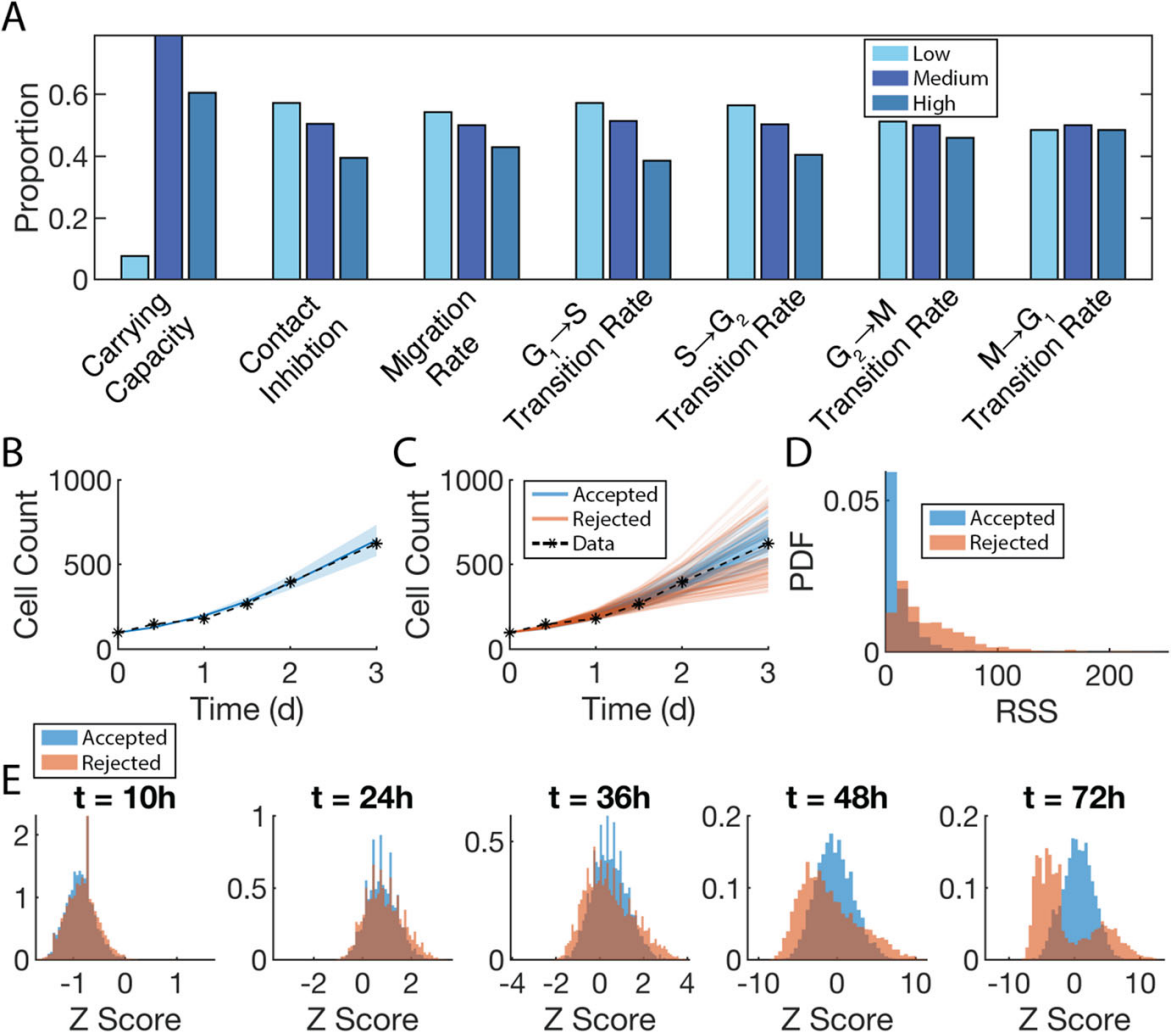
Evaluating SMoRe ParS performance in constraining ABM parameter space. **a** Proportions of each of the seven ABM parameters that SMoRe ParS accepted in the low (light blue bars), medium (dark blue bars), and high (teal bars) ranges. **b** Comparison of ABM-generated averaged cell count time-course (blue curve) using SMoRe ParS-inferred parameters versus experimental data (black asterisks and dashed curve). Shaded region shows ± SD in ABM simulations. **c** A sampling of individual time-courses generated by the ABM using accepted (blue curves) versus rejected (red curves) parameters plotted together with the experimental data (black asterisks and dashed curve). **d** Residual sum of squares (RSS) distributions obtained from SMoRe ParS-accepted (blue PDF) and rejected (red PDF) parameters. **E** Z-scores of individual ABM simulations, sorted by accepted or rejected, using the experimental mean and standard deviation at those time points. Y-axis is normalized to a PDF (Color figure online)

Implementing the final step in SMoRe ParS, we arrive at an inferred region of ABM parameter space that maps to the experimental data. The proportions of each of the seven ABM parameters that SMoRe ParS accepted in their low, medium, and high ranges (see Methods Sect. 2.5) are shown in Fig. 7a. Notably, a higher proportion of medium and high values for the carrying capacity are admitted, while median and low values are accepted more for the contact inhibition, migration rate, and three of the four transition rates. For the transition from M to G1, about the same proportion of low, medium, and high parameter values are accepted.

Next, we evaluate the effectiveness of our method by comparing the experimental data with the mean ABM output generated using SMoRe ParS-inferred parameter values (Fig. 7b). The dotted line represents the experimental data, and the dark blue line represents the mean ABM output, with the light blue shadow representing one standard deviation. Mean ABM cell count time courses show excellent agreement with experimental data, suggesting we have successfully identified the desired subset of ABM parameter space. It is important to note that we did not select these ABM parameters by direct comparison with the data, but by using the SM as an interlocutor and implementing the SMoRe ParS algorithm. A sampling of individual time-courses of accepted (blue) versus rejected (orange) ABM parameters are shown in Fig. 7c. The ‘accepted’ curves cluster around the mean of the experimental data, whereas the ‘rejected’ curves are more spread out and form two distinct groupings, one below the experimental mean and one above it, neither of which match the experimental data. Overall, this shows that in general, our method is able to constrain ABM parameter space from given experimental data.

We can evaluate how well SMoRe ParS selects/rejects the correct subsets of ABM parameters by computing the RSS of the mean trajectory from each ABM parameter vector. The distributions of these RSS values distinguished by accepted/rejected shows a lower median for the accepted distribution and a much longer tail for the rejected distribution (Fig. 7d). That is, SMoRe ParS is more likely to accept parameters the better they fit the data. We can further evaluate the contribution of each time point to this overall RSS score by looking at the z-scores of the cell counts at these time points (Fig. 7e). At early times, there is little to distinguish between the accepted and rejected ABM vectors, with both either under-estimating ($t=10$h) or mostly over-estimating ($t=24$h) experimental data (see Supplementary Figure S3). As time progresses ($t=36$h and $t=48$h), accepted ABM vectors clearly cluster more tightly around a z-score of 0—the mean of the experimental data—than rejected vectors. By the end of the simulation ($t=72$h) the z-scores of the rejected vectors split into a bimodal distribution spread out away from the experimental mean, whereas the z-scores of the accepted vectors are in a unimodal distribution that is closer to the mean.

### SMoRe ParS Yields Comparable Results to the Direct Method

**Fig. 8:**
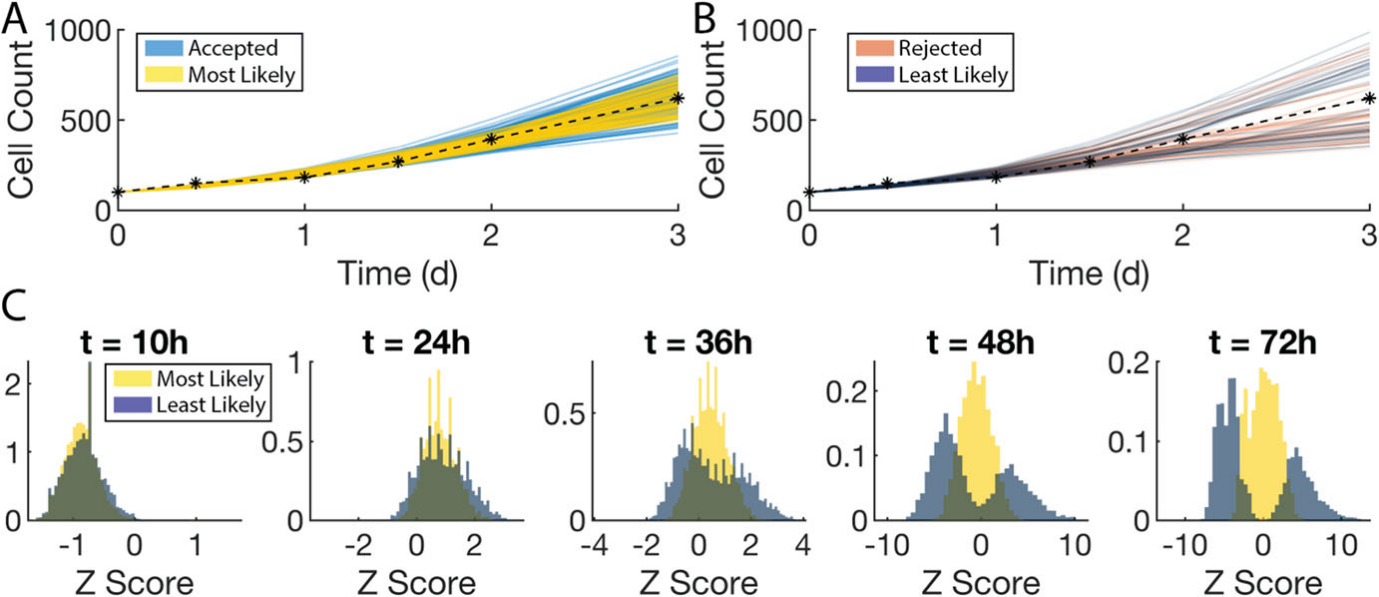
Comparing SMoRe ParS performance to the direct method. **a** A sampling of individual time-courses generated by the ABM using SMoRe ParS-accepted (blue curves) versus most likely (maize curves) parameters plotted together with the experimental data (black asterisks and dashed curve). **b** Similar to A but comparing SMoRe ParS-rejected (red) and least likely (dark blue). **c** Z-scores of individual ABM simulations, sorted by most likely (maize) and least likely (dark blue), using the experimental mean and standard deviation at those time points. Y-axis is normalized to a PDF (Color figure online)

To validate our method for ABM parameterization, we compare how well ABM simulations match the experimental data when using SMoRe ParS-inferred parameters versus parameters inferred by direct comparison (see Methods Sect. 2.7). We plot sample ABM-generated trajectories using SMoRe ParS-accepted parameters (light blue curves) and those using the ‘most likely parameters’, that is, from the direct method (yellow curves) (Fig. 8a). Both sets of trajectories are close to the experimental data mean with SMoRe ParS trajectories showing slightly larger variances at later time points. We also consider the SMoRe ParS-rejected versus ‘least likely’ parameters and samplings of their trajectories (Fig. 8b). Both sets can be seen to diverge from the experimental mean starting at around $t={2}\hbox { d}$. For further details on how we compared simulated and experimental time series, see Supplementary Information Section S4.

Finally, we evaluate the direct method’s performance in reproducing experimental data at each time point by looking at the z-scores of the cell counts at these time points (Fig. 8c). As in the case of SMoRe ParS (Fig. 7e), the z-scores of the least likely vectors split into a bimodal distribution spread out away from the experimental mean, whereas the z-scores of the accepted vectors are in a unimodal distribution that is closer to the mean.

### Using Practical Identifiability Information about SM Parameters Improves SMoRe ParS’ Performance

We conclude our analysis of the control study by evaluating the importance of using practically identifiable combinations of SM parameters, as informed by experimental data, in the final step of SMoRe ParS. We accomplish this by first arriving at experimental data-informed SM parameters in two distinct ways, taking $\Phi \subset {\mathbb {R}}^3$ be the Cartesian product of the experimental data-informed 95% confidence intervals for each SM parameter and $\Psi \subset {\mathbb {R}}^3$ be the union of the experimental data-informed practically identifiable combinations of SM parameters. We next implement SMoRe ParS to constrain ABM parameter space using these two sets of SM parameters, as explained in Methods Sect. 2.7. Finally, we compare how well the experimental data is matched by (averaged) ABM output generated using the two resultant sets of ABM parameters.

**Fig. 9:**
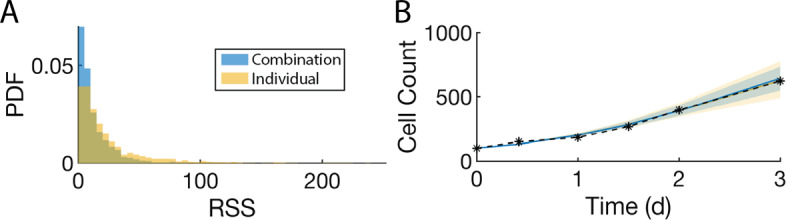
Comparing acceptance in SMoRe ParS with and without using practically identifiable parameter combinations. **a** Distribution of RSS values accepted using parameter combinations (blue) or not (yellow). **b** Distribution of mean trajectories of ABM parameters selected using combinations (blue) or not (yellow). Shaded area indicates ± SD (Color figure online)

The RSS distributions plotted in Fig. 9a illustrate how well ABM output generated using each of these parameter spaces matches the experimental data. These distributions were calculated from the weighted RSS between averaged ABM realizations and the experimental data. As can be seen, ABM parameters inferred using practical identifiability of SM parameters tend to have lower RSS values and thus better fits (blue bars) as compared to ABM parameters inferred simply using 95% confidence bounds on SM parameters (yellow bars). The rightward shift in RSS values of this superset of ABM parameters comes from an increased variance at later time points (Fig. 9b). The variance of cell count curves from the superset parameter profiles (yellow lines) is more spread out whereas the variance of the cell count curves from the more constrained parameter profiles (blue lines) is closer to the mean values. Thus, SMoRe ParS yields a better constrained ABM parameter set when we account for SM parameter identifiability properties.

### Multidimensional Experimental Data and the ABM Output Successfully Constrain Surrogate Model Parameter Space in the Treatment Case

**Fig. 10:**
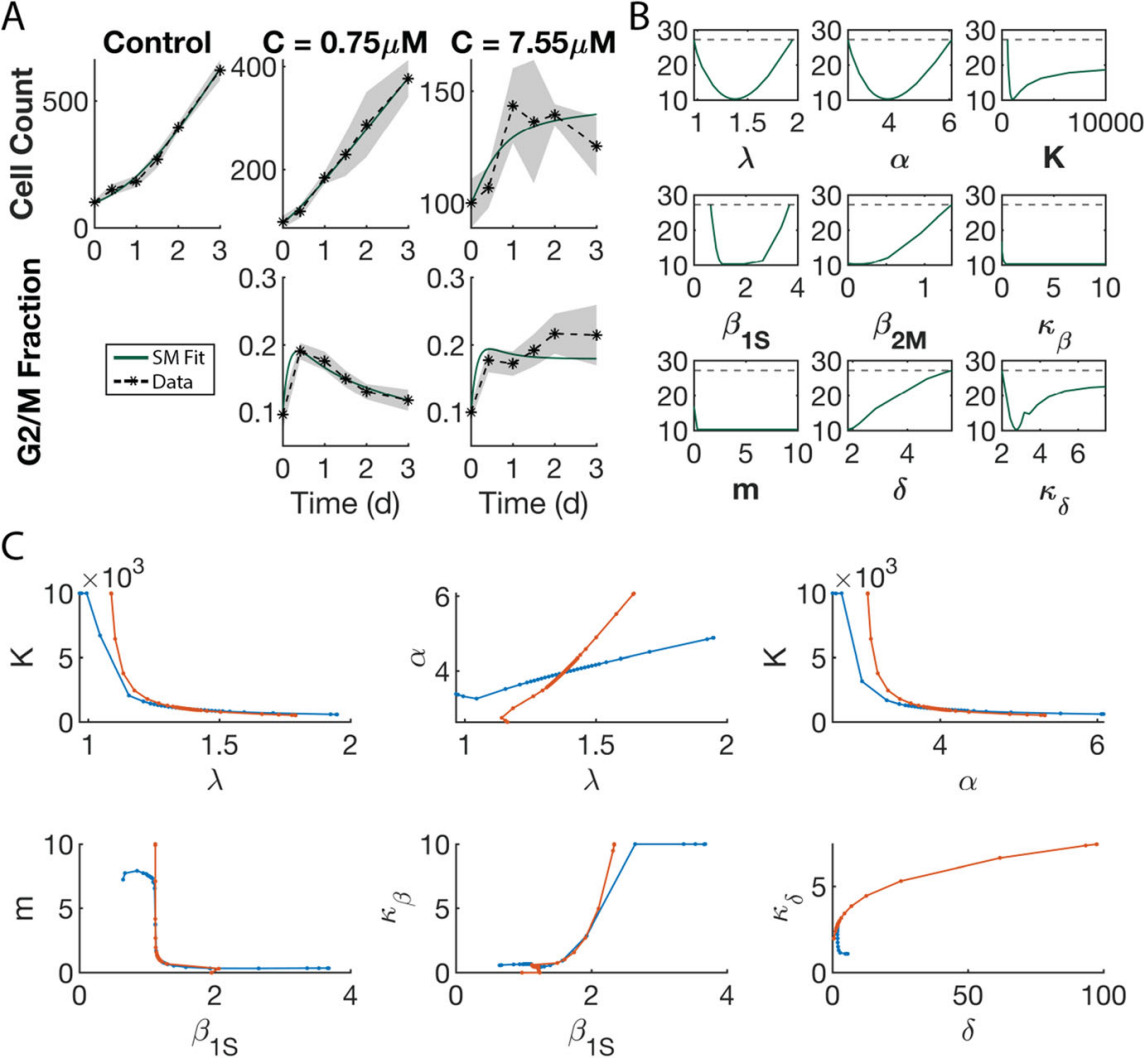
Constraining SM parameters from experimental data. **a** SM best fit to experimental data. **b** Profiles of all SM parameters from fitting to experimental data. **c** Exploring parameter combinations of the SM fit to the experimental data. In each plot, blue (red) curves correspond to the parameter along the *x*-axis (*y*-axis) being profiled (Color figure online)

Having demonstrated that SMoRe ParS performs well when constraining a high-dimensional ABM parameter space with simple, unidimensional data, we now test how well it performs when using multidimensional experimental data. By multidimensional data, we mean data of multiple ‘types’. For instance, time-courses of tumor volumes in xenograft assays, potentially testing multi-drug/multi-dose anti-cancer therapy, or time-courses together with end-point data such as a dose-response or survival curves. Our SM for the treatment case (Eq. 5) has nine free parameters that are fit to data comprising tumor growth inhibition time-courses for three different drug doses (0 $\upmu$M, 0.75 $\upmu$M, and 7.55$\upmu$M) and cell cycle distribution time courses for the two nonzero drug doses. Best fits are plotted together with experimental data in Fig. 10a. A practical identifiability analysis reveals that the treatment-specific parameters $\alpha , \lambda , \beta _{1\,S}, \beta _{2\,M}$ and $\delta$ are identifiable in most instances, while the remaining SM parameters are identifiable from above or below (Fig. 10b). To uncover any hidden combination structure between parameters, we plot pairwise relationship curves between parameters (Fig. 10c). These curves are generated by plotting non-profiled parameter estimates versus the profiled parameter. Blue/red curves correspond to the parameter along the *x*-/*y*-axis being profiled, respectively. Largely coincident curves suggest a pairwise combination structure, as is the case for *m*-$\beta _{1S}$, $k_{\beta }$-$\beta _{1S}$ and $k_{\delta }$-$\delta$. On the other hand, $\lambda$, $\alpha$ and *K* are a simultaneous combination, with no clear pairwise combination structure. We remark that only those parameter profiles are shown where a practically identifiable combination exists.

**Fig. 11:**
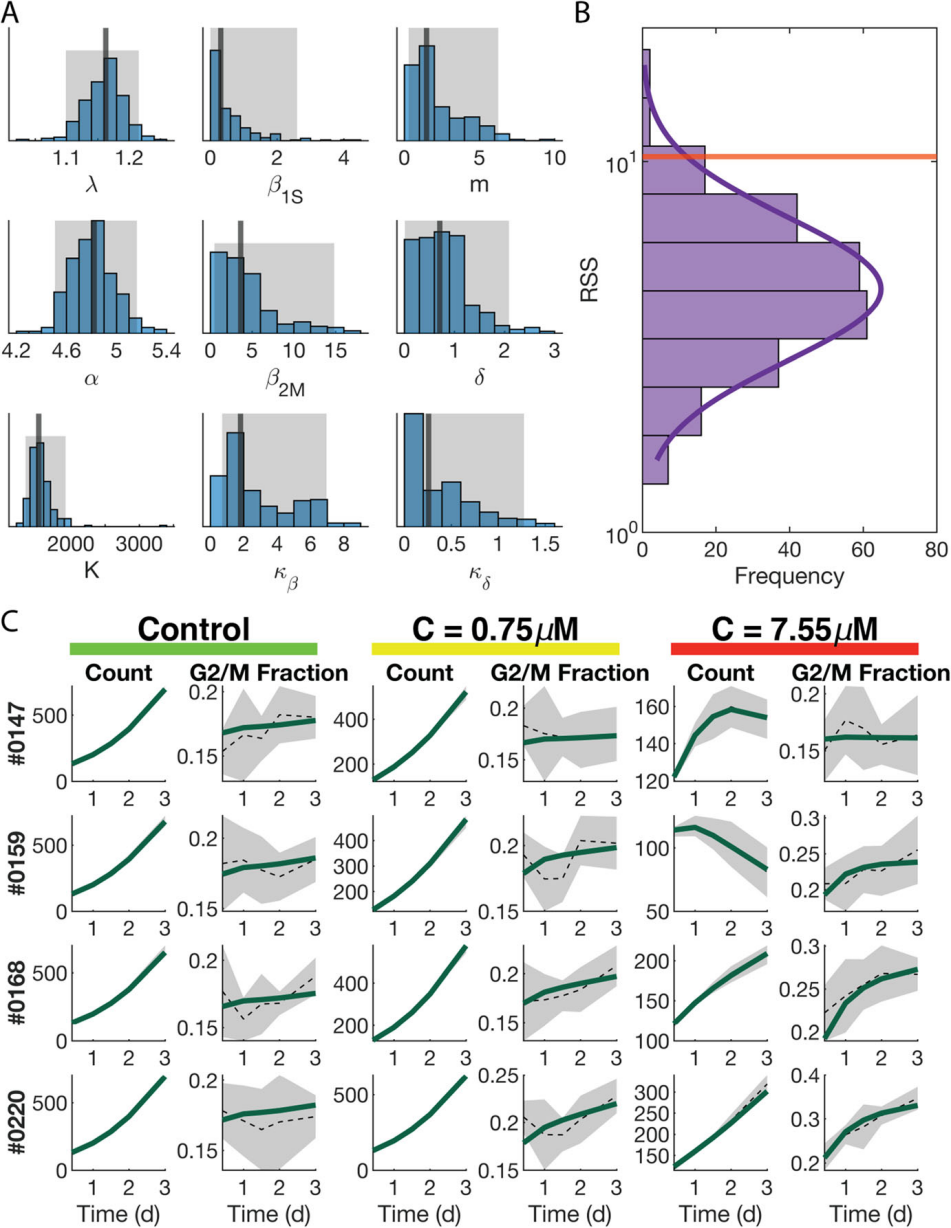
Fitting the SM to each ABM parameter vector output. **a** Distribution of best fit values of SM parameters to the ABM output. Vertical line indicates median value. Shaded area indicates the interquantile range. **b** Distribution of RSS values from the best fit of the SM to ABM output at each ABM parameter vector. Orange line indicates the RSS value for the best fit of the SM to the experimental data. **c** Sampling of four ABM parameter vectors (rows) and the fit of the SM to the two experimentally measured outputs for each experimental condition. Dark green curves represent the SM fit. Mean ABM output shown in dashed black. Shaded area is ± SD of ABM output (Color figure online)

Next, we fit these treatment parameters from the SM to averaged ABM output generated at each of the 3$\times$3$^5$ sampled parameter vectors (see Methods Sect. 2.5). The resulting distributions for the SM parameters $\lambda$, $\alpha$, and *K* (Fig. 11a) are bell-shaped, resembling those obtained from the control study (see Fig. 5a–c), suggesting that these parameters have a well-constrained range of values that effectively encapsulates the entire ABM output variability. The distributions on the other 6 SM parameters are less clear-cut, suggesting a higher degree of uncertainty. The RSS values of SM parameters fit to ABM output follow a log-normal distribution (Fig. 11b), and are smaller than the RSS from fitting the SM to experimental data (Fig. 11b, orange line). The time series trajectories for the treatment SM demonstrate good fits to the ABM output for both, total tumor size, and G2/M fraction across the different dosing strategies (Fig. 11c). We finally profile each SM parameter using the ABM output at each ABM parameter vector (see Supplementary Figure S5). The criterion for accepting an ABM parameter follows the same procedure as outlined in the control case (Sect. 3.5).

### SMoRe ParS Infers High Dimensional ABM Parameter Spaces that Successfully Reproduce the Multidimensional Experimental Data

**Fig. 12:**
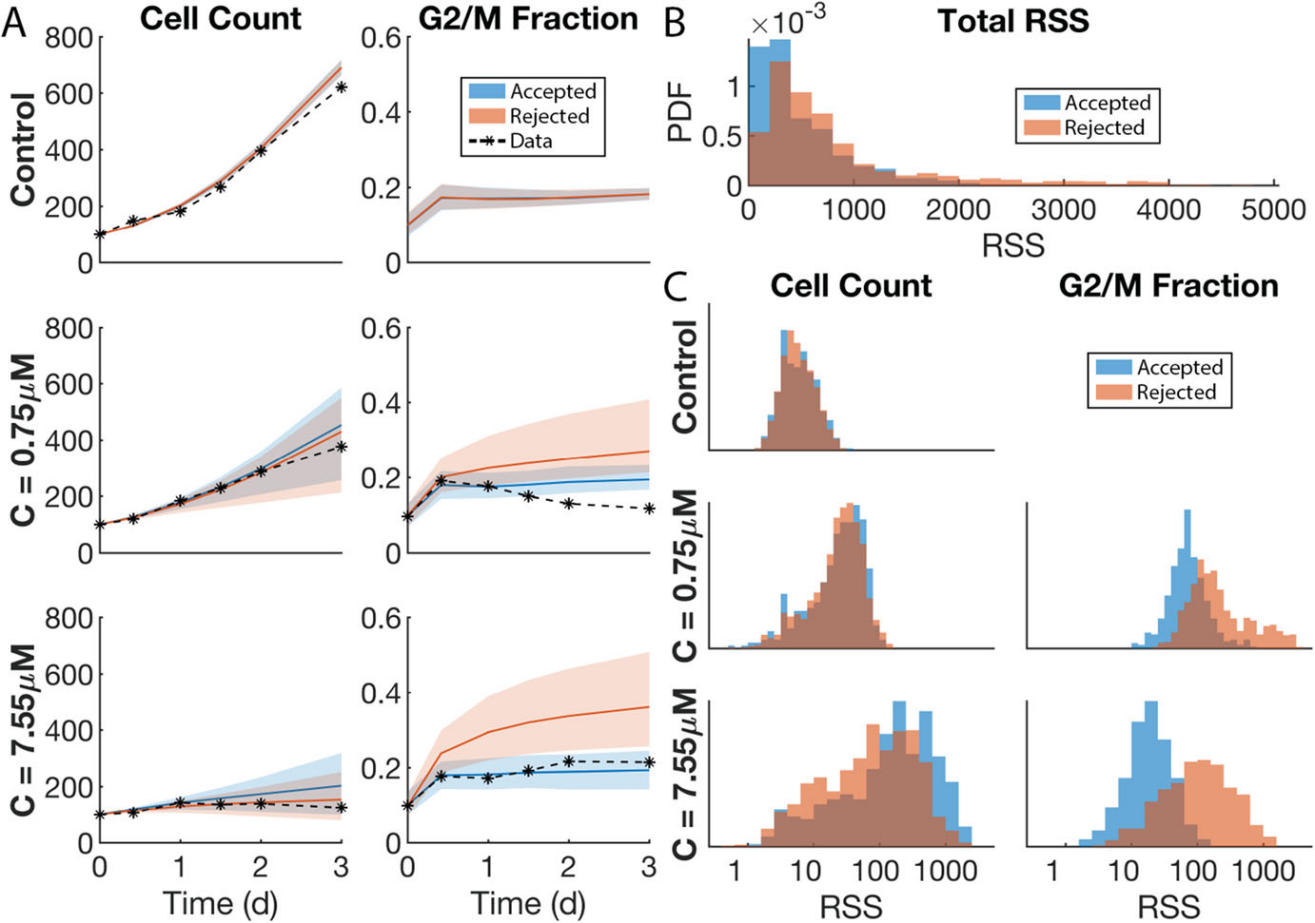
Evaluating SMoRe ParS performance in constraining ABM parameter space with multidimensional data. **a** Comparison of ABM-generated averaged time series using SMoRe ParS-inferred parameters versus experimental data (black asterisks and dashed curve). SMoRe ParS-accepted (-rejected) parameters shown in blue (red). Shaded region shows ± SD in ABM simulations. **b** Residual sum of squares (RSS) distributions obtained from SMoRe ParS-accepted (blue PDF) and rejected (red PDF) parameters across all experimental conditions and time series. **c** Breakdown of contributions to total RSS in **b** from each experimental condition and time series. *x*-axis limits are preserved across all plots (Color figure online)

We evaluate the effectiveness of our method by comparing the mean ABM output at the SMoRe ParS-inferred parameter values, with the multidimensional experimental data (Fig. 12a). As in the control study, ABM generated averaged cell count time courses for the no treatment case (Fig. 12a, first panel) show excellent agreement with experimental data. For the treatment cases, averaged time-courses generated using accepted (blue) ABM parameters fit G2/M fractions well, whereas those generated using rejected (orange) ABM parameters are farther above the experimental time-courses than the accepted curves (Fig. 12a, rows two and three, second column). Interestingly, there is little to distinguish total cell count time-courses in the treatment case generated using accepted (blue) or rejected (orange) ABM parameters, with both curves overestimating the experimental data (Fig. 12a, rows two and three, first column). Indeed, the rejected parameters time-courses are marginally closer to the experimental data than the accepted parameters time-courses, although it should be noted that the experimental data are contained within one standard deviation of the accepted parameters time-courses.

Overall, SMoRe ParS constrains ABM parameter space successfully even in this multidimensional data case, as evidenced by the RSS distributions of accepted versus rejected ABM parameter vectors (Fig. 12b). These distributions were calculated by summing the weighted RSS between averaged ABM realizations and each distinct experimental data set. The RSS distribution of the accepted parameters (blue bars) is clustered near zero with a lower median and shorter tail than that for the rejected parameters (orange bars).

Individual contributions to the total RSS from fitting each data set are shown in Fig. 12c. There appears to be little difference between RSS values for the total cell counts coming from rejected and accepted parameters in the control and 0.75 $\upmu$M dose cases. For the 7.55 $\upmu$M dosing schedule, the distribution of rejected RSS values are slightly shifted left compared to the accepted RSS values (Fig. 12c, bottom left panel). This corresponds to how close the respective mean cell count time-courses are to the experimental data (Fig. 12a, bottom left panel). In contrast, RSS values coming from G2/M fraction time-courses for the rejected parameters are clearly shifted to the right compared to the accepted parameters (Fig. 12c, right column), demonstrating that the SMoRe ParS-constrained ABM fits these data well.

**Fig. 13:**
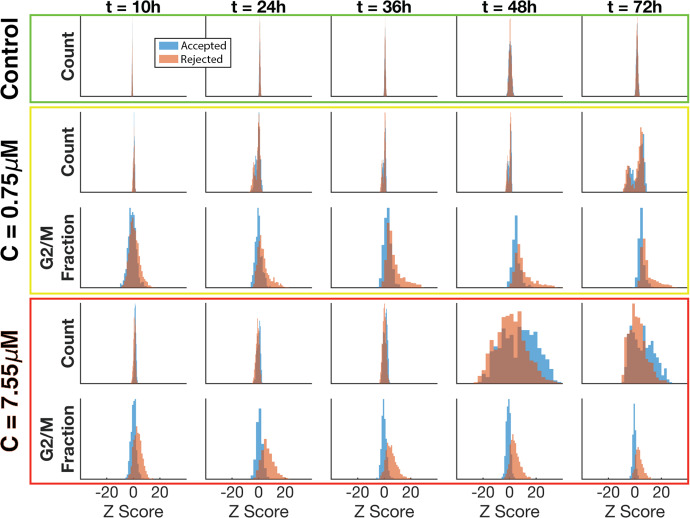
Normalized residuals of ABM output compared to the experimental data across all time points, experimental conditions, and time series

As we did for the control case (Fig. 7e), we calculate the z-scores of the ABM output for both accepted and rejected parameters, comparing count and G2/M fraction across all experimental conditions using the experimental data as the population distribution (Fig. 13). Recall, the experimental data did not quantify cell cycle state in the control arm. For the control data, there is little difference between the accepted and rejected data across all times (green box). For the first treatment condition, there is little difference between the accepted and rejected count data on hour 10. Between hour 24 and 48, the count data starts to look bimodal and by hour 72, there is a clear bimodal distribution with the rejected count values shifted to the left (yellow box). For the first treatment condition, the z-scores of the G2/M fraction show a normal distribution around 0 for the accepted values and a right-skewed distribution for the rejected values. The skewing to the right for the rejected values increases as the number of hours increases (yellow box).

Therefore, the accepted values are closer to the mean experimental values than the rejected values and are thus a better fit. For the second treatment condition, there is little difference between the accepted and rejected count data on hours 10 through 36. On hours 48 and 72, the rejected values seem to skew to the right and are slightly left of center. There is a bimodal distribution with the accepted count values centered around 0 (red box). For the second treatment condition, the z-scores of the G2/M fraction show a tight normal distribution around 0 for the accepted values and a wider normal distribution shifted slightly to the right for the rejected values (red box). Therefore, the accepted values are closer to the mean experimental values than the rejected values and are thus a better fit. We remark that using a different set of control ABM parameters determined by randomly sampling from the set of accepted parameters from the control study resulted in similar distributions of z-scores (see Supplementary Figures S6 and S7).

## Discussion

The urgent challenges posed by health and environmental crises demand immediate attention and effective solutions. With the aim of advancing human health and minimizing potential harm, data is being generated at an unprecedented scale, and in multidimensional forms including -omics data, biochemical pathways, web and social media data and integrated digitized administrative databases, to name a few (Luo et al. 2016). There is a critical need for time- and cost-efficient strategies to analyze and interpret this data, and agent-based models are increasingly being called upon to generate reasonable, testable hypotheses from such complex data sets (Castro et al. 2020; Jain et al. 2022).

In this study, we developed a novel computational framework that constrains high dimensional ABM parameter space to multidimensional real-world data. This framework builds on our recently published approach, Surrogate Modeling for Reconstructing Parameter Spaces (SMoRe ParS) (Jain et al. 2022), that leverages explicitly formulated surrogate models to link ABMs and experimental data. The resultant method encodes within it uncertainty quantification of ABM parameter values, this uncertainty stemming from both, stochasticity in ABM simulations, and error/noise in experimental data. Therefore, SMoRe ParS gives the user a *distributional sense* of ABM parameter space as constrained by data.

We began by extending the SMoRe ParS algorithm to constrain a high dimensional parameter space of an easy-to-simulate ABM, using unidimensional data. ABM output generated using SMoRe ParS-inferred parameters was seen to be in excellent agreement with the data, demonstrating our method’s accuracy. We next compared the results of our methodology with those obtained when using ABM input parameters estimated directly from the experimental data. This is only computationally feasible in this situation, with an easy-to-simulate ABM. Both sets of ABM output yielded similar degrees of fits to the data, thereby validating our approach. We remark that even in this easy-to-simulate case, direct estimation of ABM parameters was only possible with a simple acceptance-rejection algorithm, from a predetermined set of input parameter values. We did not perform a formal parameter estimation, for example, a gradient descent method that would require the user to run thousands to millions of ABM simulations. In contrast, a particular strength of our method, which we demonstrated in Jain et al. (2022), is that it allows for the exploration of ABM parameter space even at points that are not directly sampled and at which ABM output was never generated.

Frequently, parameter estimation for predictive models lacks a comprehensive identifiability analysis (Eisenberg and Jain 2017). Instead uncertainty in estimated parameters is often assessed using 95% confidence intervals derived from the data, resulting in independent (ranges of) values for each parameter. This approach overlooks potential interdependencies among parameters, resulting in disparate parameter combinations that may not align well with the data. Here, we illustrated this potential pitfall by comparing experimental data with ABM output generated using practically identifiable combinations of surrogate model parameters versus ignoring this identifiability structure. Our results suggest that SMoRe ParS yields best results when using practical identifiable information on surrogate model parameters contained within the experimental data.

Scholarly works presenting computationally efficient methods to connect ABMs with multidimensional data are scarce. Cess and Finley (Cess and Finley 2023) offer a creative new computational approach that applies neural networks to parameterize an ABM of tumor growth with spatially resolved imaging data. While their strategy enables a quantitative comparison of tumor images and ABM simulations, it is computationally expensive. Additionally, incorporating temporal data or data at varying scales into this method is challenging. Renardy et al. (2020) employ a hybrid sampling-estimation approach to parameterize their discrete and stochastic network-based model of the spread of COVID-19. Specifically, they generated ABM output at 500 parameter sets and minimized a cost function to arrive at a ‘best-fit’ parameter set. However, this approach suffers from the same drawbacks as our aforementioned direct method for ABM calibration: (1) we are restricted to only those parameter combinations at which ABM output was generated and cannot fine-tune parameters further, which is especially an issue if the parameter samples were widely spaced to begin with; and (2) increasing the number of sampled parameter combinations can become computationally unfeasible in a complex ABM.

Here, we offer a new tool for researchers in need of methods to connect ABMs to data sets that involve measures of various types or at biological scales that are beyond the feasibility of ABMs to simulate. Having adapted SMoRe ParS to constrain high dimensional ABM parameter spaces with *uni*dimensional data, we next customized it to constrain high dimensional ABM parameter spaces with *multi*dimensional data, taking examples from tumor cell growth inhibition assays to demonstrate our method. ABM-generated averaged cell count time courses for the pre-treatment case were found to be in excellent agreement with experimental data, as were the averaged time courses for G2/M fractions in the treatment case, when using SMoRe ParS-inferred parameters. As expected, using rejected parameters yielded poor matches to these data. In contrast, accepted and rejected parameters matched the experimental cell count time-courses for the treatment case equally well. For example, the rejected mean of total cell counts was closer to the data than the accepted mean, but both were still within one standard deviation of one another, and of the data. Taken together, these results imply that cell cycle fraction is the distinguishing factor, and SMoRe ParS succeeds in capturing these mechanistic details. In the context of cancer treatment, such details can matter greatly since platinum-based drugs such as oxaliplatin are frequently combined with anti-mitotic drugs such as taxanes in treating solid tumors (Pavlidis and Pentheroudakis 2012). Taxanes are cell-cycle specific and predicting optimal combination dosing strategies successfully is predicated on knowing cell cycle distributions resulting from exposure to one or the other drug accurately (Eisenberg and Jain 2017). This example demonstrates that SMoRe ParS effectively infers high-dimensional ABM parameter spaces, leading to the accurate reproduction of multidimensional experimental data.

The work presented here is a significant step towards operationalizing SMoRe ParS for broader use, in more general settings. At the same time, our results suggest several avenues for future work as we seek to further improve our method’s performance and applicability. For instance, we will examine the impact of surrogate model selection on SMoRe ParS’ performance. A single surrogate model may not fit all ABM output equally well, as was observed with our treatment model (data not shown). Equally, an optimal surrogate model may exist that can fit all ABM output within a user-specified error threshold. However, this prospect might be overlooked due to the present trial-and-error approach in SMoRe ParS, where each surrogate model improvement is systematically evaluated. We plan to address these issues by allowing for multiple surrogate models, potentially arrived at through machine learning techniques such as equation learning (Nardini et al. 2021) or BINNs (Biologically-informed neural networks) (Lagergren et al. 2020).

Through the analysis presented here, we also highlight the fact that multidimensional data presents unique, but solvable challenges in general, and for SMoRe ParS in particular. For example, the individual contributions to the total RSS from fitting each data set shown in Fig. 12c sum up to the RSS distributions of accepted versus rejected ABM parameter vectors in Fig. 12b. This raises the question: should individual contributions to the RSS from fitting each data set be given equal weight or should data-set specific weights be applied? While machine learning approaches for arriving at a better surrogate model could potentially improve the overall fit, the answer to this question will ultimately differ depending on the model application being studied and the types of data available. Users of SMoRe Pars should decide which data set(s) are most important based on the specific questions they want to use their model to address.

In conclusion, our work introduces a robust and scalable computational framework, SMoRe ParS, designed to explore the uncertainty within multidimensional parameter spaces of ABMs representing complex biological phenomena. By enabling the effective incorporation of noisy and sparse real-world data, this platform extends the potential of ABMs to unveil hidden and counter-intuitive mechanistic features hidden in complex data sets. These revelations can have profound implications for predicted outcomes and planned interventions, accentuating the utility of mathematical modeling in advancing our understanding of complex systems.

### Supplementary Information

Below is the link to the electronic supplementary material.

Supplementary file 1 (pdf 901 KB)

## Data Availability

The experimental data used in this study can be found in the references listed.

## Code availability

Code for simulating the various models and implementing SMoRe ParS for these models will be made available on request.
