# Supplementary material for: Connecting Agent-Based Models with High-Dimensional Parameter Spaces to Multidimensional Data Using SMoRe ParS: A Surrogate Modeling Approach
Source: Bull Math Biol. 2023 Dec 30;86(1):11. doi: 10.1007/s11538-023-01240-6 (PMC10757706; doi:10.1007/s11538-023-01240-6)
Supplement: Supplementary file 1 — (pdf 901 KB) [file 11538_2023_1240_MOESM1_ESM.pdf]

SUPPLEMENTARY MATERIAL

S1 ABM Algorithm

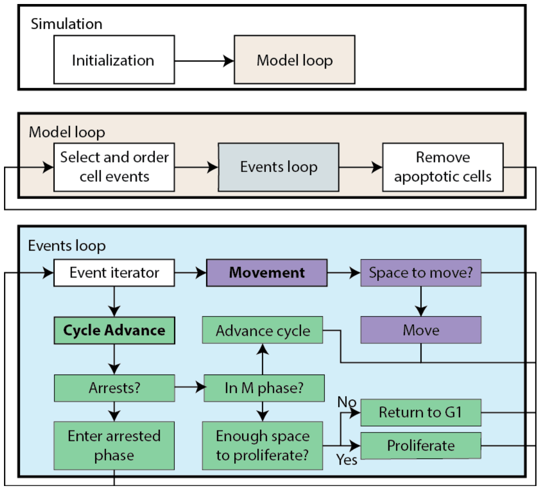

FIGURE S1. Flowchart detailing the ABM algorithm.

S2 ABM Parameterization using SMoRe ParS

In Section 2.7 in the main text, we describe a geometric interpretation for how SMoRe ParS accepts or rejects an ABM parameter. Figure 2 in the main text shows an example of a parameter being accepted when only using the confidence intervals and when using practical identifiable combinations. In Figure S2 below, we show an example of an ABM parameter vector that is rejected both with and without using parameter combinations. The parameter vector is rejected in this case because the data-derived and ABM output-inferred confidence bounds of one of the surrogate model parameters (K) do not overlap.

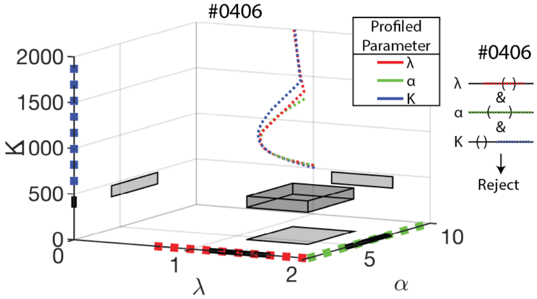

FIGURE S2. Geometric representation of when SMoRe ParS rejects an ABM parameter vector. Parameter vector #0406 is rejected because the data-derived and ABM output-inferred confidence bounds of one of the surrogate model parameters, K, do not overlap.

S3 Evaluating SMoRe ParS Performance in Constraining Parameter Space at Lower and Higher Time Points

In order to better demonstrate how cell counts generated using SMoRe ParS-constrained ABM parameters compare to experimental data at lower time points, we plot the initial two time points on separate plots in Figure S3 below. The overall fits are shown in Figure 10C of the main text. At time $t = 10\,\text{h}$ ABM-generated cell counts (both accepted and rejected) using SMoRe ParS initially under-predicts the experimental data (Figure S3A). At time $t = 24\,\text{h}$ most, but not all, of the ABM-generated cell counts (both accepted and rejected) over-predict the experimental value (Figure S3B). At the subsequent time-courses (shown in Figure 6C of the main text), the accepted ABM-generated cell counts accurately predicts the subsequent time-course values.

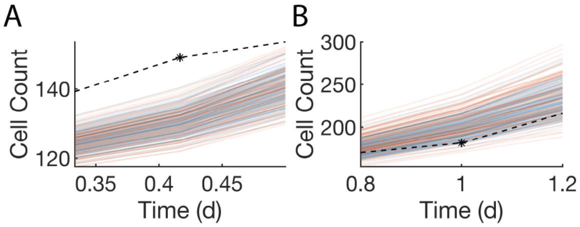

FIGURE S3. Sample ABM output colored by accepted (blue) or rejected (red) near time $t = 10\,\text{h}$ (A) and $t = 24\,\text{h}$ (B). Experimental data shown with dashed line and '*' at the observed data point.

S4 Statistical Model to Compare Simulated and Experimental Time Series

The experimental data includes observations at five time points after $t = 0$: $t = 10, 24, 36, 48, 72\,\text{h}$. We assume that the observations, X_i, at these different time points, t_i, in the experimental data are independent and normally distributed. That is,

$$X_i \sim \mathcal{N}(\mu_i, \sigma_i), \quad 1 \leq i \leq 5 \tag{S1}$$

where μ_i and σ_i are the experimentally observed mean and standard deviation, respectively, at time t_i. Thus, the likelihood of a trajectory is related (see below) to the weighted sum of squares of the residuals at the time points where the weights are given by the experimental standard deviations. We refer to this weighted sum as the residual sum of squares (RSS), and it is given by

$$\mathrm{RSS}_j = \sum_{i=1}^{5} \left(\frac{x_{ij} - \mu_i}{\sigma_i} \right)^2 \tag{S2}$$

where $(x_{ij})_{i=1}^{5}$ is the time series observation from experiment j.

At times, it is useful to convert these RSS values into likelihood values, LL:

$$\begin{aligned} \mathrm{LL}_j &= \sum_{i=1}^{5} -\frac{1}{2} \left(\frac{x_{ij} - \mu_i}{\sigma_i} \right)^2 - \log\left(\sigma_i \sqrt{2\pi}\right) && \text{(S3)} \\ &= -\frac{1}{2}\mathrm{RSS}_j - 5\log\left(\sqrt{2\pi}\right) - \sum_{i=1}^{5} \log\left(\sigma_i\right) && \text{(S4)} \\ &= -\frac{1}{2}\mathrm{RSS}_j + C && \text{(S5)} \end{aligned}$$

Note that this constant is identical within a given experimental condition.

These log-likelihood values, however, only make sense relative to one another, and so we create relative likelihoods, RL_j, by exponentiating the log-likelihoods and normalizing by the largest:

$$\mathrm{RL}_j = \frac{\exp\left(\mathrm{LL}_j\right)}{\max\limits_k \exp\left(\mathrm{LL}_k\right)} = \frac{\exp\left(-\frac{1}{2}\mathrm{RSS}_j\right)}{\exp\left(-\frac{1}{2}\min\limits_k \mathrm{RSS}_k\right)} \tag{S6}$$

In this way, a relative likelihood of 0.5 can be interpreted as "50% as likely as the most likely." We use the identical formulation when comparing to the ABM output.

When we want to assign an RSS, LL, or RL to an ABM parameter vector, we compute these quantities for the mean trajectory across 6 realizations of the model with those parameters. When considering the oxaliplatin experiments, we compute mean trajectories under all three experimental conditions and use the sum of the resulting RSS values as the RSS value for the ABM parameter vector.

We apply this methodology to the RSS values from the control model. We plot the distribution of these relative likelihoods grouped by rejected and accepted (Figure S4A). We also plot the distributions for all parameters, the most likely, and the least likely to provide a reference. We observe that only ABM parameters less than 35% as likely as the most likely even have a chance of being rejected.

When we bin the ABM parameters by their likelihood and look at the proportion of SMoRe ParS-accepted parameters within each bin (Figure S4B), we see that this proportion grows with the likelihood. This indicates that SMoRe ParS is more likely to accept a parameter when that parameter produces a lower (higher) RSS (likelihood) value.

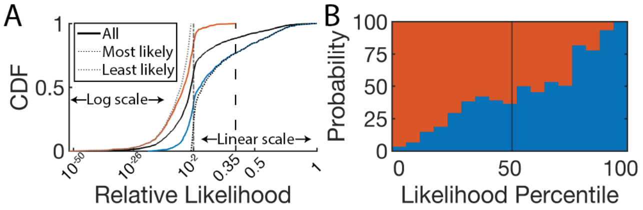

FIGURE S4. (A) Cumulative density functions of relative likelihoods of ABM parameter vectors as estimated by RSS values, grouped by accepted (blue) and rejected (red). The same is shown for the most likely (gray dashed), least likely (black dashed), and all (black solid) for comparison. Vertical line indicates the switch from log scale (left) to linear scale (right). (B) Probability, as a percentage, of accepting an ABM parameter vector within binned percentiles of their likelihoods as estimated by RSS values. Vertical line indicates the percent of rejected ABM parameter vectors.

S5 Parameter Profiles for the Chemotherapy Case

We again use the profile-likelihood method to arrive at 95% confidence bounds for each of the SM parameters being fit to (averaged) ABM output at each ABM parameter vector (Figure S5). We find that λ and α are identifiable, just as when we fit these parameters to experimental data (Figure 10B, main text). For a given SM parameter, its 95% confidence bounds correspond to discrete points on the upper and lower 95% confidence hypersurfaces that lie over 7-dimensional ABM parameter space.

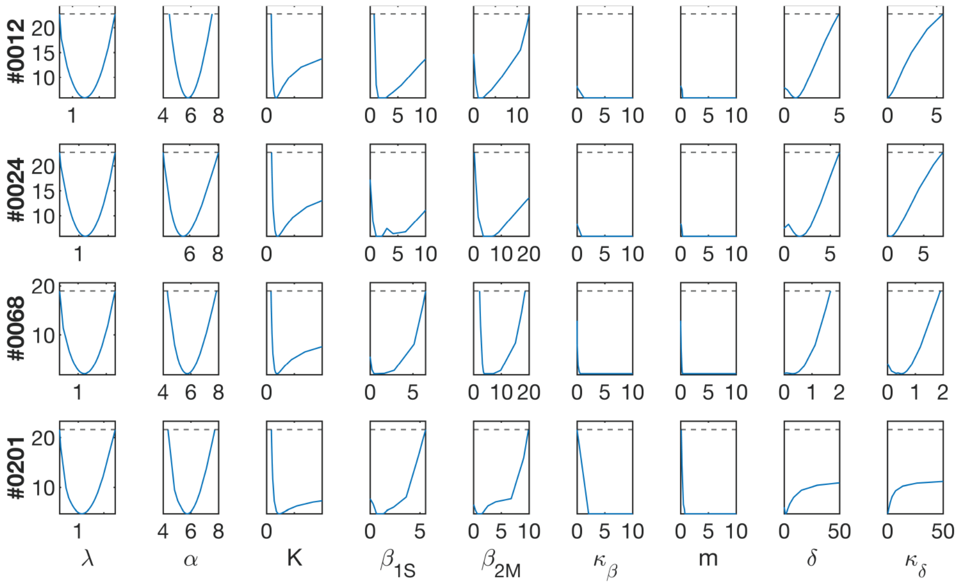

FIGURE S5. SM parameter profiles at a sampling of ABM parameter vectors.

S6 SMoRe ParS Infers High Dimensional ABM Parameter Spaces that Successfully Reproduce the Multidimensional Experimental Data, Using an Alternative Set of Control Parameter Values

In Section 3.7 of the main text, we evaluated the effectiveness of our method by comparing the mean ABM output at the SMoRe ParS-inferred parameter values, with the multidimensional experimental data. For this, we randomly sampled from the set of accepted ABM parameter vectors from the control study, and held them fixed whilst estimating ABM treatment parameter vectors. To eliminate the possibility that SMoRe ParS' performed well simply because we happened on a fortuitous choice of ABM control parameters, we repeat the SMoRe ParS pipeline using a different randomly selected set of control ABM parameters. As can be seen in Figure S6, similarly good fits are obtained to the experimental data. Further, Figure S7 shows similar distributions of z-scores broken down by time point and experimental condition.

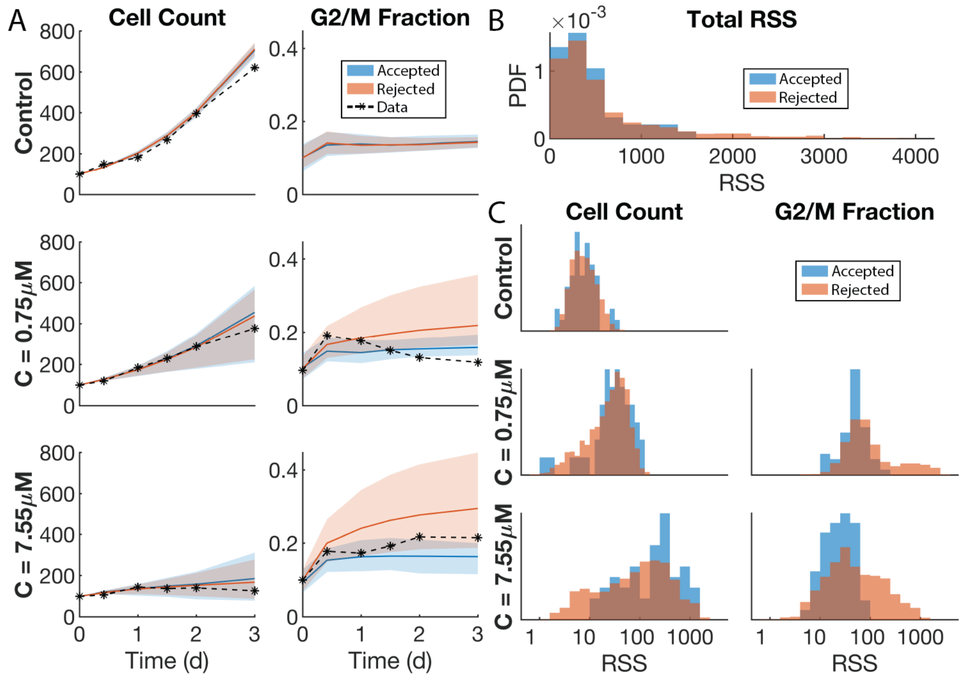

FIGURE S6. Evaluating SMoRe ParS performance in constraining ABM parameter space with multidimensional data. (A) Comparison of ABM-generated averaged time series using SMoRe ParS-inferred parameters versus experimental data (black asterisks and dashed curve). SMoRe ParS-accepted (-rejected) parameters shown in blue (red). Shaded region shows ± SD in ABM simulations. (B) Residual sum of squares (RSS) distributions obtained from SMoRe ParS-accepted (blue PDF) and rejected (red PDF) parameters across all experimental conditions and time series. (C) Breakdown of contributions to total RSS in B from each experimental condition and time series. x-axis limits are preserved across all plots. Compare to Figure 11 of main text.

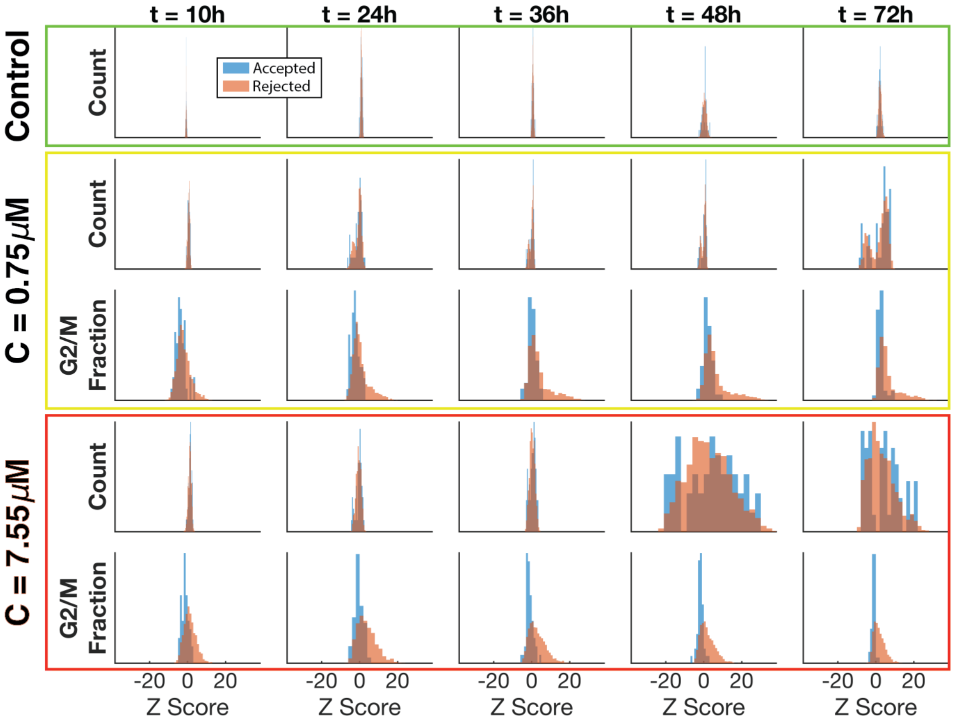

FIGURE S7. Normalized residuals of ABM output compared to the experimental data across all time points, experimental conditions, and time series. Compare to Figure 12 of the main text.
